# Discovery of CD4^+^ T cell–recognized *B*. *pertussis* antigens that reduce airway colonization

**DOI:** 10.1172/jci.insight.199234

**Published:** 2026-09-08

**Authors:** Mohamed M. Shamseldin, Jesse M. Hall, Griffin M. Lawrence, Gabrielle M. Hernandez, Yue Liu, Alexa R. Aubrey, Eva K. Verzani, Rajendar Deora, Amy Lovett-Racke, Jennifer G. Abelin, Purnima Dubey

**Affiliations:** 1 Department of Microbial Infection and Immunity and; 2 Department of Microbiology, The Ohio State University, Columbus, Ohio, USA.; 3 Department of Microbiology and Immunology, Faculty of Pharmacy, Helwan University–Ain Helwan, Helwan, Egypt.; 4 Broad Institute of Massachusetts Institute of Technology and Harvard, Cambridge, Massachusetts, USA.; 5 Infectious Diseases Institute, The Ohio State University Wexner Medical Center, Columbus, Ohio, USA.; 6 Department of Neuroscience, The Ohio State University, Columbus, Ohio, USA.; 7 Pelotonia Institute for Immuno-Oncology, James Comprehensive Cancer Center, Columbus, Ohio, USA.

**Keywords:** Immunology, Infectious disease, MHC class 2, Proteomics, Vaccines

## Abstract

Despite widespread vaccination, *Bordetella pertussis* (*Bp*) cases are resurging globally. Although CD4^+^ T cells are known to be essential for sustained protection, the antigens they recognize are not fully characterized, hindering vaccine refinement. Using immunopeptidomics, bioinformatics, and functional T cell assays, we identified high-affinity epitopes from reference and clinical *Bp* strains presented on MHC-II I-A^b^. A subset of these epitopes stimulated systemic and mucosal CD4^+^ T cells of mice immunized with heat-killed *Bp*, and peripheral blood T cells from humans vaccinated with the whole-cell pertussis vaccine. Mice immunized with a subunit vaccine comprising two recombinant proteins identified in our screen were subsequently challenged with *Bp*. Bacterial burden was nearly eliminated from the lower respiratory tract and significantly reduced in the upper respiratory tract. Th1/Th17-polarized CD4^+^ tissue-resident memory T cells (Trms) were induced in nasal and pulmonary tissues. Depleting memory CD4^+^ T cells before challenge abolished protection, confirming that antigen-specific CD4^+^ T cells are critical for clearing *Bp* from the respiratory tract. Our integrated antigen identification and T cell assay approach revealed previously untested *Bp* antigens that elicit protective CD4^+^ T cell–mediated immunity, suggesting that incorporating them into new vaccines may help curb the resurgence of pertussis.

## Abstract 2

An unbiased approach identified *Bordetella pertussis* T-cell antigens that, in an experimental subunit vaccine, induced protective T cell responses and reduced respiratory bacterial burden

## Graphical abstract

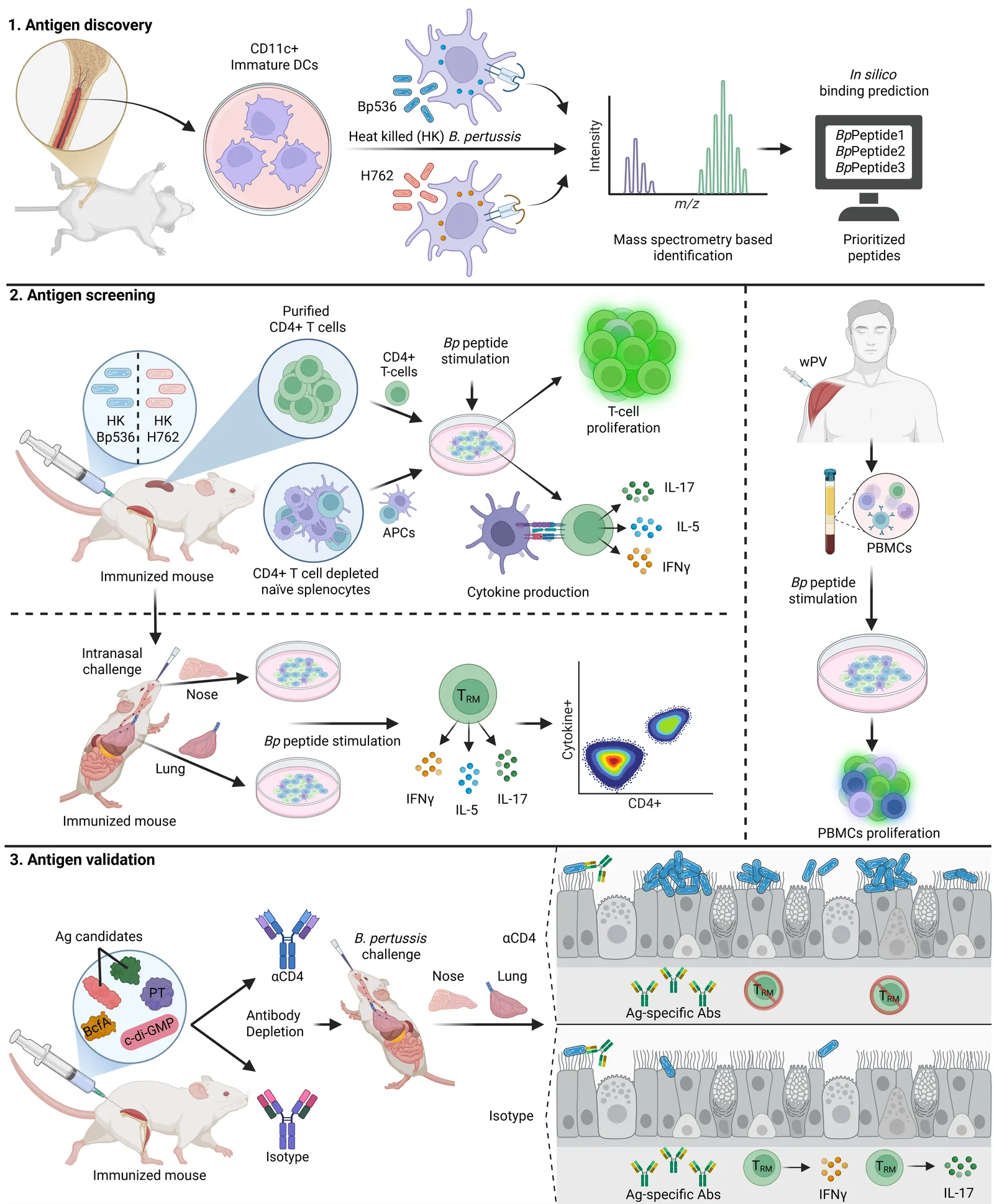

## Introduction

Despite widespread vaccination efforts, whooping cough (pertussis) — caused by the Gram-negative bacterium *Bordetella pertussis* (*Bp*) — is experiencing a global resurgence (1). Whole-cell pertussis vaccines (wPVs), used since the 1940s, combated severe disease, and eliminated bacterial burden in the upper and lower respiratory tract, thereby preventing transmission to vulnerable populations (2). However, wPVs are highly reactogenic (3, 4), prompting the development of less reactogenic acellular pertussis vaccines (aPVs). These vaccines comprise 1–5 *Bordetella* proteins adjuvanted with alum, and have been used since the late 1990s, particularly in the Western world. Systemic immunization with aPV elicits neutralizing antibodies that prevent severe disease and bacterial colonization of the lungs (5). However, aPV-generated immunity is short-lived, requiring frequent boosters (5).

Th1/Th17-polarized T cell responses and tissue-resident CD4^+^ memory T cells (Trms) are critical for sustained protection against infection (6, 7). While wPV elicits Th1/Th17 responses and mucosal immunity, aPVs elicit Th2-polarized T cell responses (8, 9) and do not generate mucosal immunity (10, 11). This dichotomy in immune responses elicited by wPVs and aPVs provides one explanation for the resurgence of *Bp* infections.

Although most *Bp* studies use the reference strain Tohama I and its derivatives, current clinical *Bp* isolates exhibit considerable genetic and phenotypic diversity compared with the laboratory strain (12, 13) with negligible or reduced expression of aPV antigens (14–16). Thus, there is a critical need to identify antigens that protect against circulating strains.

Despite the acknowledged importance of CD4^+^ T cell responses in providing sustained protection against *Bp* infection (17), the antigens that generate these T cell responses are not fully characterized. Previous work to identify immunogenic *Bp* proteins has largely focused on antibody targets (18–21). To devise more effective next-generation pertussis vaccines that elicit mucosal immunity, it is critical to identify the *Bp*-derived immunogenic proteins that are recognized by CD4^+^ T cells. To achieve this goal, we undertook an untargeted mass spectrometry screen to identify the MHC-II I-A^b^–presented immunopeptidome from a laboratory strain, Bp536 (22, 23), and a clinical *Bp* isolate, H762 (24, 25). We discovered that a set of MHC-II–bound peptides derived from unique bacterial source proteins were recognized by systemic CD4^+^ T cells and tissue-resident CD4^+^ T cells that were recruited to the nose and lungs of *Bp*-challenged mice. These MHC-II peptides were also recognized by T cells of wPV-immunized humans. An acellular vaccine containing 2 of these parent proteins elicited T cell responses in mice that cleared *Bp* bacterial burden from the lungs and reduced colonization of the nose in immunized mice. Depletion of both tissue-resident and systemic CD4^+^ T cells prior to *Bp* challenge in immunized mice impaired bacterial clearance, indicating that CD4^+^ T cells are the primary protective mechanism induced by the experimental vaccine.

Herein, we identified *Bp* antigens that elicit protective CD4^+^ T cell responses by integrating mass spectrometry–based immunopeptidomics with functional T cell assays. These findings lay the groundwork for developing next-generation acellular pertussis vaccines capable of inducing mucosal immunity and curbing the resurgence of pertussis.

## Results

### The B. pertussis MHC-II immunopeptidome.

We undertook an untargeted discovery approach to identify immunogenic proteins from *Bp* that are presented by MHC-II molecules on antigen-presenting cells (APCs) (26). We used the *Bp* laboratory reference strain Bp536 (22, 23), isolated in the 1950s, and a clinical strain, H762 (24, 25), isolated in 2011, as prototypes to compare the conservation and divergence of *Bp* MHC-II immunopeptidomes.

**Figure 1:**
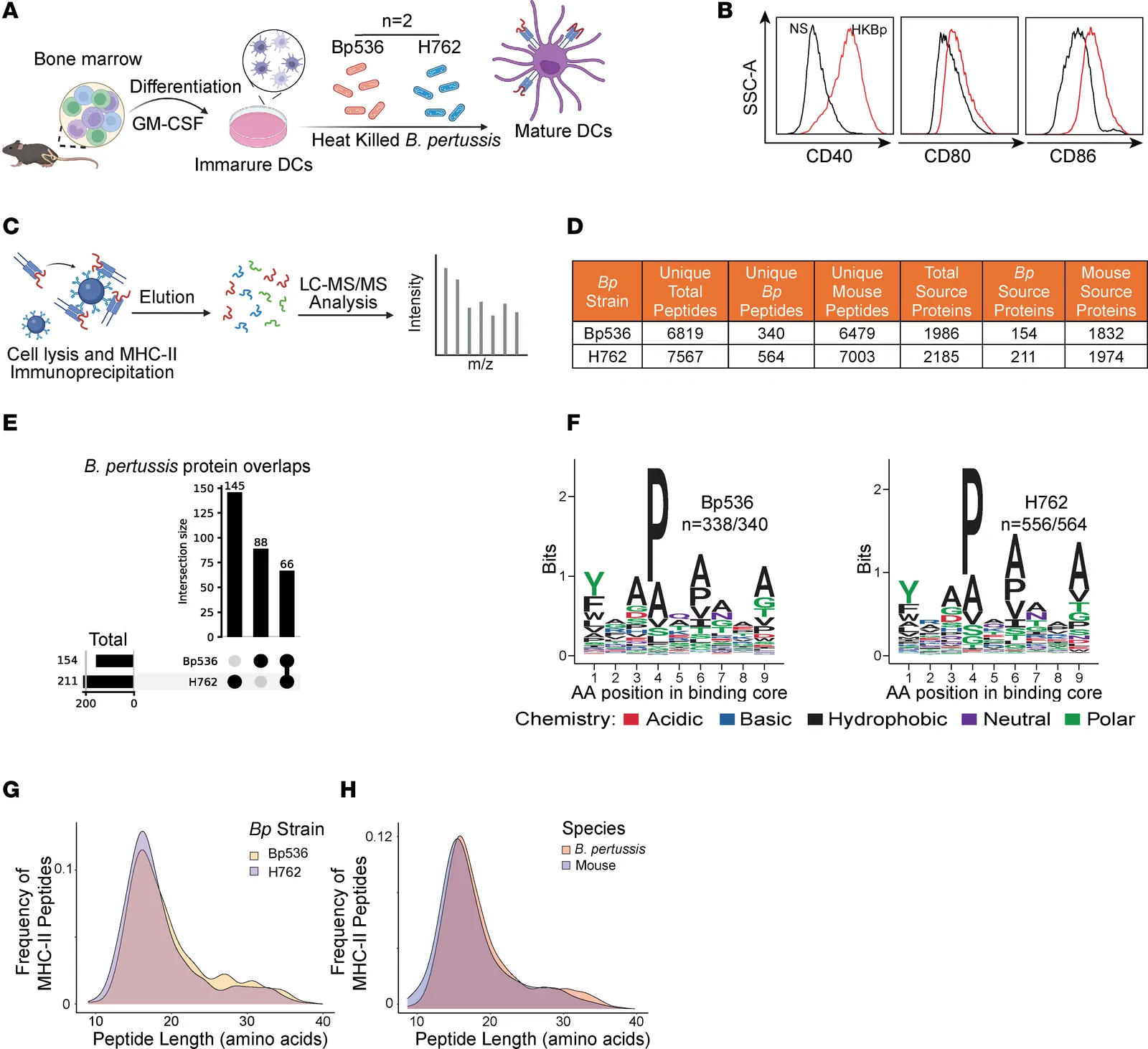
IP and mass spectrometry analysis of *Bp* peptides presented on I-A^b^. (**A**) BMDC differentiation and treatment with heat-killed *Bp* (HKBp). (**B**) Costimulatory molecule expression on HKBp-treated BMDCs. Black lines, unstimulated BMDCs; red lines, BMDCs treated with heat-killed Bp536. (**C**) Schematic of IP and LC-MS/MS analysis. (**D**) Summary of *Bp* and murine peptides identified by mass spectrometry. (**E**) UpSet plot showing the overlap in *Bp* source proteins identified by mass spectrometry. The overlap of source proteins between strains was determined based on full-length protein sequences. (**F**) *Bp* peptides immunoprecipitated from I-A^b^ of Bp536- and H762-treated BMDCs display the canonical I-A^b^ motif. (**G** and **H**) Frequency of *Bp* peptides by strain (**G**) and frequency of *Bp* peptides by species (**H**).

Murine bone marrow–derived dendritic cells (BMDCs) were differentiated (27) (Figure 1A), and immature BMDCs were treated with heat-killed Bp536 or H762 (HKBp) and tested for activation by flow cytometry. BMDCs were defined as CD11c^+^ I-A^b^–high cells, and upregulation of the costimulatory molecules CD40, CD80, and CD86 indicated activation (Figure 1B). The I-A^b^ MHC-II molecules were immunoprecipitated using anti–I-A^b^ antibody Y3P (28, 29) from pooled batches of 3 × 10^8^ to 6 × 10^8^ treated BMDCs per replicate (Figure 1C). MHC-II–associated peptides from 2 biological replicates for each strain were acid-eluted and analyzed by liquid chromatography/tandem mass spectrometry (LC-MS/MS). The resulting peptide sequences were compared with the normal murine proteome and the *Bp* proteome to identify species-specific peptides (Figure 1D). This analysis identified 6,819 unique peptides from 1,986 source proteins in the Bp536 IP and 7,567 unique peptides from 2,185 proteins in the H762 IP (Supplemental Table 1; supplemental material available online with this article; https://doi.org/10.1172/jci.insight.199234DS1). Of these, 340–564 unique *Bp* peptides were derived from 154–211 *Bp* source proteins. The UpSet plot (Figure 1E) shows the overlap of source proteins of MHC-II peptides in each immunopeptidomics analysis, indicating which source proteins were unique to each strain and which source proteins were shared in the immunopeptidome of BMDCs treated with heat-killed Bp536 and H762. An approximately 22% overlap between *Bp* class II MHC source proteins was observed between strains based on full-length protein sequences. The number of *Bp* proteins identified in each IP replicate is shown in Supplemental Figure 1A. As expected, the pathogen-derived MHC-II peptide sequences from both *Bp* strains (Figure 1F) and the murine peptide sequences (Supplemental Figure 1B) matched the previously reported I-A^b^ binding motif (30). The *Bp* peptides from both strains (Figure 1G) and the murine peptides (Figure 1H) were also of the expected lengths.

### Selection of predicted high-affinity MHC-II epitopes.

Memory T cells are reactivated by T cell receptor recognition of peptide-MHC complexes on APCs (31, 32). High-affinity and/or highly abundant peptides trigger T cell precursors more effectively than low-affinity or less abundant epitopes (33, 34). Thus, we used the consensus algorithm in the Immune Epitope Database (IEDB) (35, 36) to calculate the predicted affinities of the Bp536- and H762-presented peptides for I-A^b^ and the human HLA-DR/DQ/DP 28-allele set that are the most frequently expressed MHC-II alleles in the human population (37, 38). We selected all unique MHC-II–bound peptides from source proteins that were shared between both IP replicates of each bacterial strain, and source proteins that were shared across bacterial strains, as the input for binding predictions. The shortest peptide from a nested set (11–22 amino acids long) was evaluated for MHC-II presentation using NetMHCpan 3.0 (39, 40). Peptides with an adjusted percentile rank ≤ 10 for I-A^b^ and at least one HLA-DR/DP/DQ allele were selected (37) (Supplemental Table 2), since a lower adjusted percentile rank indicates putative MHC-II presentation. We hypothesized that these peptides would be preferentially recognized by T cells from immunized mice. This collection encompassed 50 MHC-II binding peptides, which were synthesized for functional testing. Of the selected peptides, 49 of 50 were the shortest sequences observed by LC-MS/MS, while peptide 50 was observed as a 32-mer and was presumed to be a precursor. Thus, a shorter sequence, predicted to bind to I-A^b^ with high affinity, was synthesized for testing (Supplemental Table 2).

### Functional characterization of immunodominant MHC-II epitopes.

**Figure 2:**
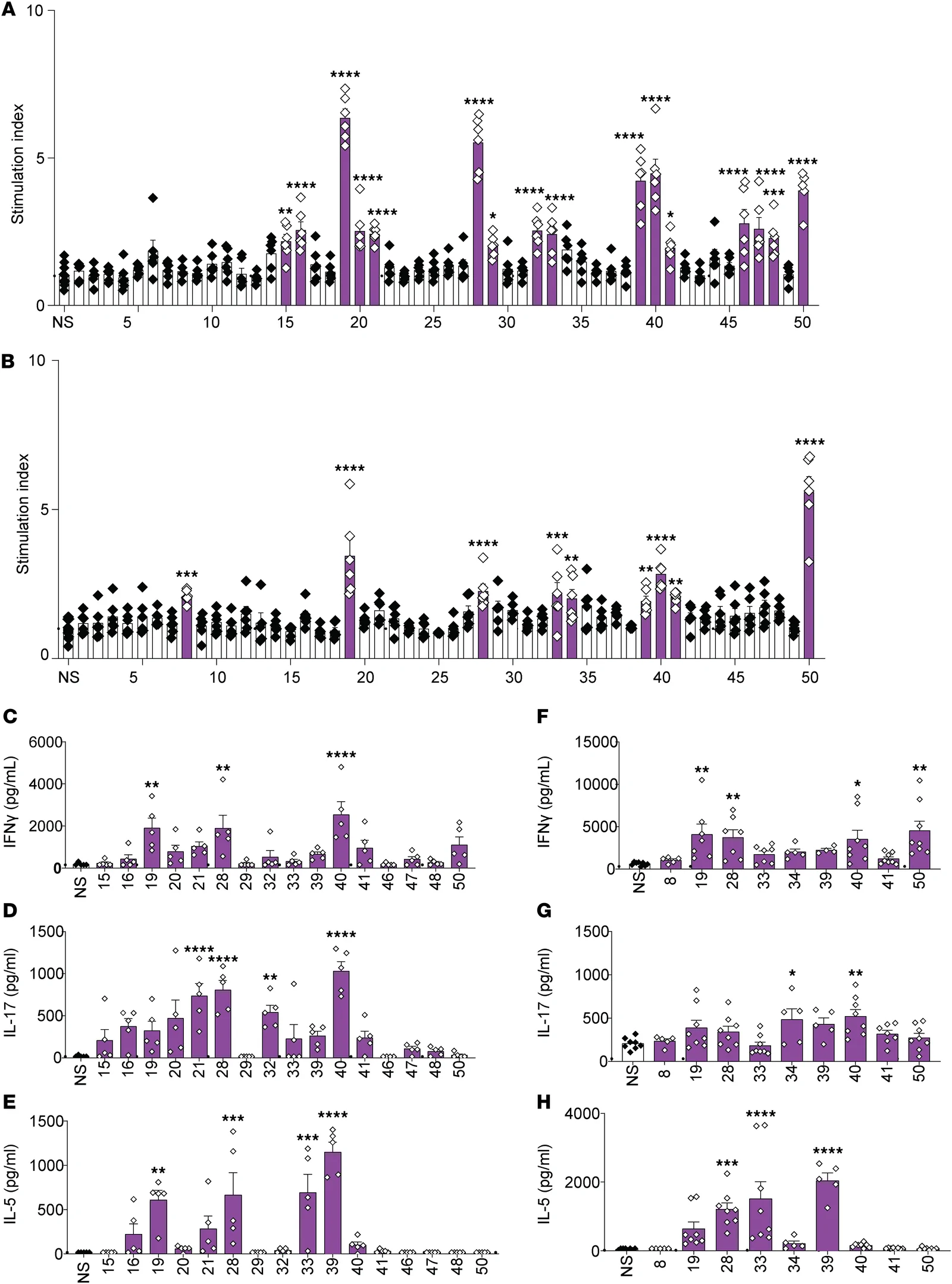
High-affinity peptides presented on I-A^b^ stimulate proliferation and cytokine production by T cells from HKBp-immunized mice. Stimulation index of HKBp-immune splenic T cells stimulated with individual peptides (peptide numbers on the *x* axis). (**A** and **B**) Splenic T cells from Bp536-immunized mice (**A**) and H762-immunized mice (**B**). Purple bars show peptides with significantly higher proliferation. (**C**–**H**) IFN-γ (**C**), IL-17 (**D**), and IL-5 (**E**) produced by Bp536-immune splenocytes, and IFN-γ (**F**), IL-17 (**G**), and IL-5 (**H**) by H762-immune splenocytes, stimulated with individual peptides were quantified by ELISA. *P < 0.05, **P < 0.01, ***P < 0.001, ****P < 0.0001 of mean ± SEM compared with nonspecific (NS) peptide control by 1-way ANOVA with Dunnett’s post hoc test. One experiment of 2 independent experiments is shown.

We used an established proliferation assay to identify immunodominant MHC-II epitopes from peptides that are recognized by memory CD4^+^ T cells. C57BL/6 mice were immunized intramuscularly (i.m.) on day 0 and on day 28 with about 1 × 10^8^ CFU of HKBp of either strain. At least 4 weeks after booster immunization, spleens were harvested and CD4^+^ T cells were purified by negative selection. CD4^+^ T cells were removed by positive selection from the spleens of naive mice, and the remaining untouched cells were used as APCs. Purity of the T cell and APC populations (>90%) was confirmed by flow cytometry (Supplemental Figure 2). Cocultures of purified CD4^+^ T cells and CD4^+^ T cell–depleted splenocytes were plated and stimulated with individual peptides from the synthesized library for 2.5 days followed by ^3^H-thymidine incorporation for 12–18 hours. Figure 2A shows that CD4^+^ T cells from Bp536-immune mice proliferated in response to 16 peptides from the library, and CD4^+^ T cells from H762-immunized mice proliferated in response to 9 MHC-II peptides (Figure 2B, purple bars).

To determine whether the peptides that elicited T cell proliferation stimulated full effector function, we cultured Bp536- and H762-immune splenocytes with each responding peptide and tested production of IFN-γ, IL-17, and IL-5 cytokines in the supernatant. Three MHC-II peptides stimulated the production of IFN-γ (Th1) (Figure 2C), 4 peptides stimulated IL-17 production (Th17) (Figure 2D), and 4 peptides induced IL-5 (Th2) (Figure 2E) from Bp536-immune spleen cells. Four MHC-II peptides stimulated IFN-γ production (Figure 2F), 2 peptides stimulated IL-17 (Figure 2G), and 3 peptides stimulated IL-5 production from H762-immune spleen cells (Figure 2H). Overall, 9 MHC-II peptides from 9 different source proteins induced proliferation and cytokine production (full effector function) from Bp536- or H762-immune CD4^+^ T cells. Five of the MHC-II peptides were recognized by CD4^+^ T cells from Bp536- and H762-immune mice, while 2 peptides (peptide 21 and peptide 32) were recognized specifically by Bp536-immune CD4^+^ T cells, and 2 peptides (peptide 34 and peptide 50) were recognized only by H762-immune CD4^+^ T cells. Together these data show that a subset of antigens presented on MHC-II are recognized by T cells from mice immunized with heat-killed bacteria.

### High-affinity MHC-II peptides generate high-avidity CD4^+^ T cells.

**Figure 3:**
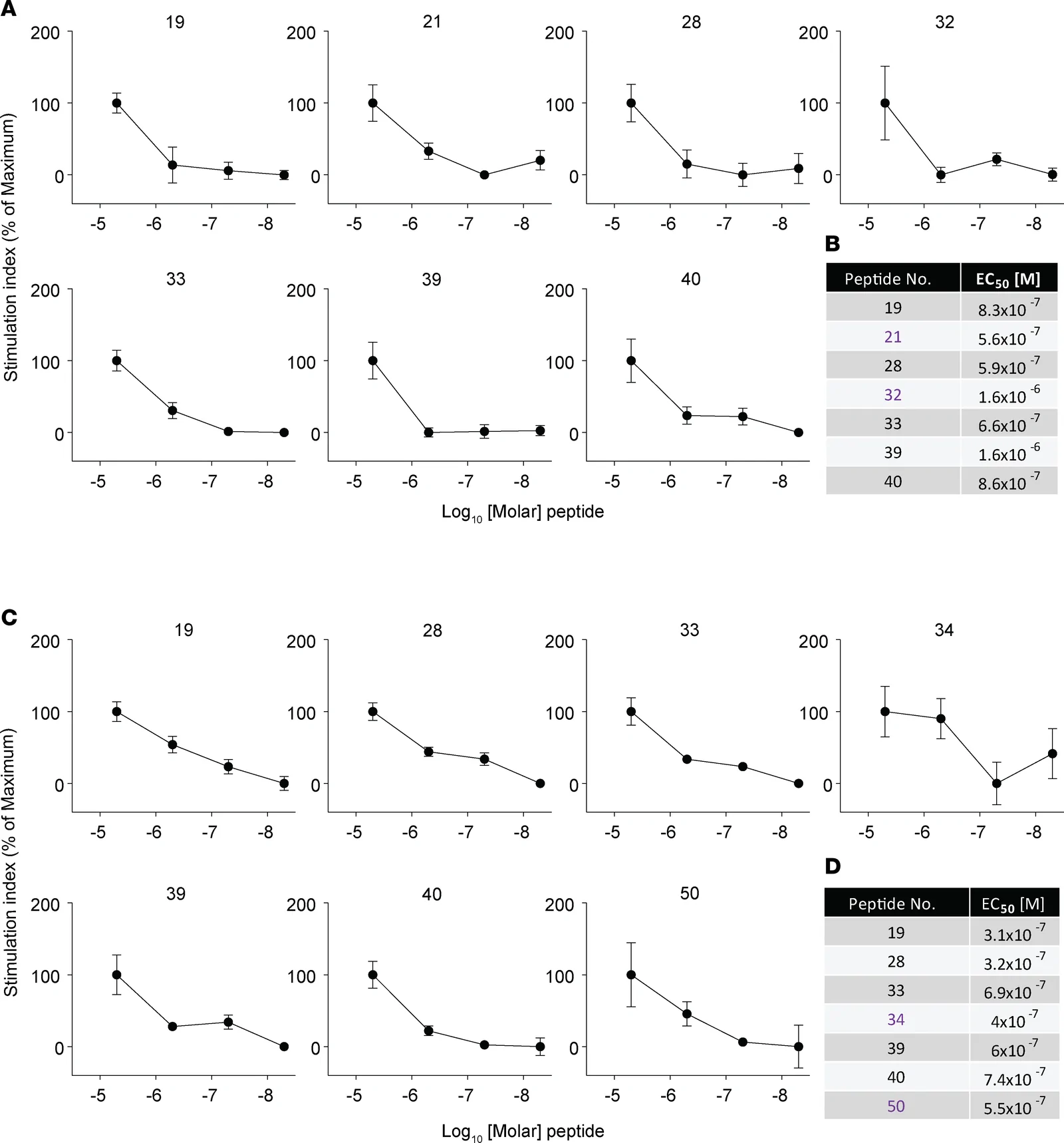
Functional avidity of stimulatory peptides. (**A**) CD4^+^ T cells from heat-killed Bp536–immunized mice stimulated with individual peptides. (**B**) Summary table of the EC_50_ of each peptide in **A**. (**C**) CD4^+^ T cells from heat-killed H762–immunized mice stimulated with individual peptides. (**D**) Summary of EC_50_ of each peptide in **B**. The peptides listed in purple in **B** and **D** were uniquely immunogenic in either Bp536-immunized or H762-immunized mice, respectively.

Functional avidity is a biological measure of the efficiency by which T cells recognize their cognate antigens (41, 42), and high functional avidity is correlated with better protection against infections and cancer (43). We measured the functional avidity of the peptides that stimulated T cell proliferation from Bp536- and H762-immunized spleen cells. Purified CD4^+^ T cells from Bp536- or H762-immune mice and CD4-depleted APCs from naive mice were stimulated with 10-fold dilutions of each peptide at concentrations ranging from 5 μM to 5 nM for 72 hours followed by ^3^H-thymidine incorporation. The stimulation index (SI) at 5 μM peptide was scaled at 100% response, and proliferation detected at lower peptide dilutions was normalized to that maximum, as previously described (44–46). After plotting of log peptide concentrations against the normalized SI, the results were fitted to nonlinear regression and used to calculate 50% of the effective concentration (EC_50_). Figure 3A shows the titration of response elicited by each peptide used to stimulate Bp536-immune T cells, with the EC_50_ of each peptide shown in Figure 3B. Figure 3C shows the titration of responses of H762-immunized T cells, with the corresponding EC_50_ of each peptide shown in Figure 3D. The peptides that were recognized by T cells from both strains and the peptides unique to each strain had similar EC_50_ values (Figure 3, B and D). Thus, all the high-affinity peptides had a low EC_50_.

### Similar affinity, abundance, and T cell avidity of immunogenic peptides.

**Figure 4:**
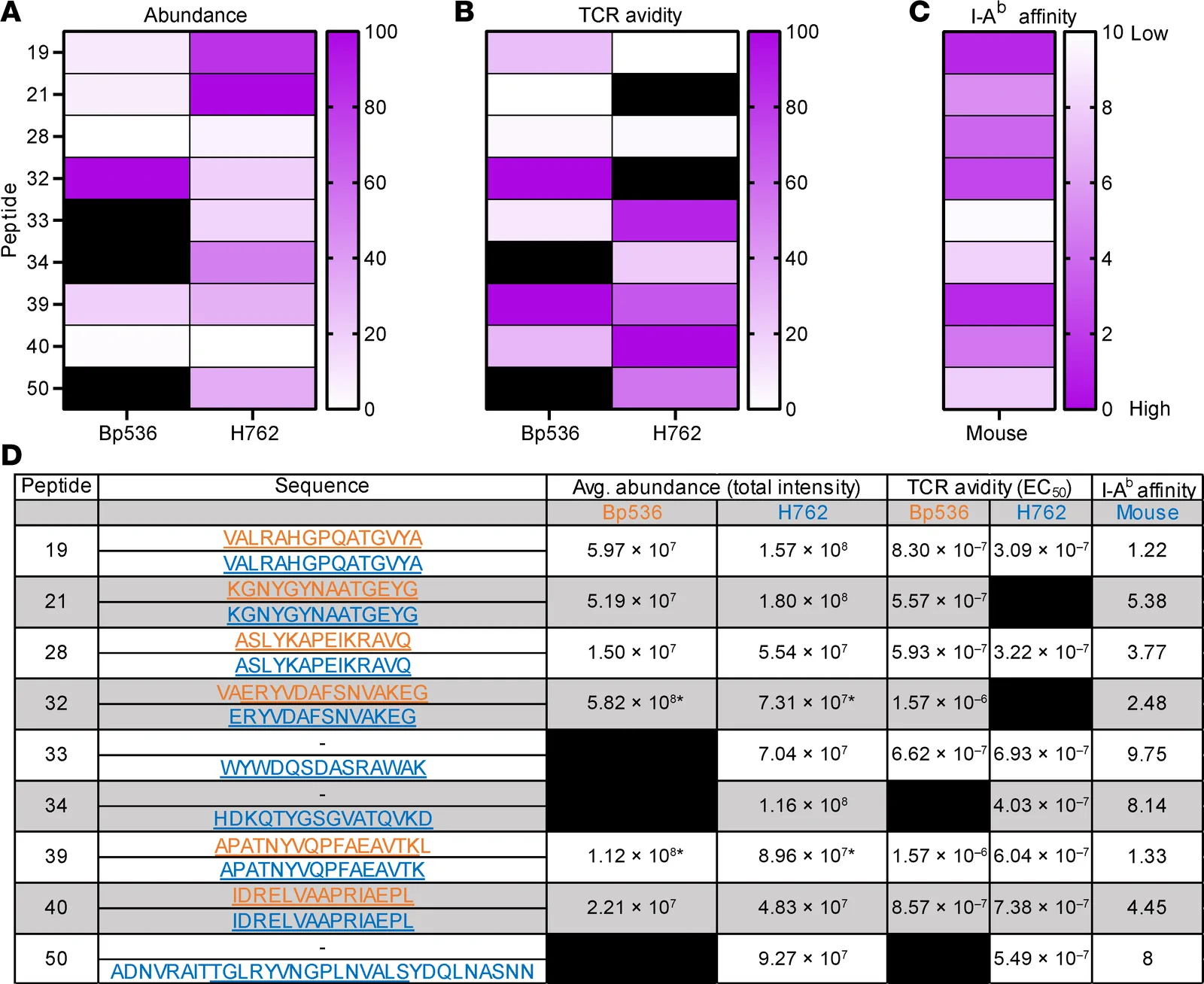
Immunoreactive peptides from Bp536 and H762 have similar abundance, predicted affinity for MHC-II, and functional avidity. (**A**–**C**) Heatmaps and summary tables show the median total peptide intensity (MS1 area under the curve), as a correlate of abundance (**A**), TCR avidity (EC_50_) for T cells stimulated by the individual peptides (**B**), and predicted MHC-II affinity (**C**). (**D**) Summary table showing stimulatory peptides from each strain with the corresponding abundance, avidity, and affinity. Peptides in orange were identified in the Bp536 immunopeptidome, and peptides in blue were identified in the H762 immunopeptidome. Data in **B**–**D** are summarized from Supplemental Table 1 and Figure 3.

Both abundance and affinity of MHC-II peptides (34, 41, 47) contribute to the generation of primary T cell responses. To determine whether the peptides that stimulated T cell responses varied in these properties, we evaluated relative peptide abundance in immunoprecipitated material using total precursor intensity (MS1 area under the curve) from the immunopeptidomics data and compared this with MHC-II affinity and functional avidity of the responding CD4^+^ T cells (Figure 4). The relative abundance (Figure 4A) and the functional avidity of MHC-II peptides isolated from the immunopeptidomes of Bp536- or H762-treated BMDCs (Figure 4B) were similar, with predicted high affinity for MHC-II (Figure 4C). These data suggest that *Bp* circulating isolates may retain immunogenic epitopes despite in vivo selection pressure and express antigens that elicit memory CD4^+^ T cell responses.

### CD4^+^ Trms recognize immunodominant peptides in the lungs and nose of Bp-challenged mice.

While it is established that Th1/ Th17-polarized CD4^+^ Trms are critical for controlling *Bp* burden in the respiratory tract (17, 48, 49), the antigens they recognize are undefined. We tested whether the MHC-II–presented antigens recognized by systemic memory T cells were recognized by CD4^+^ Trms in the nose and lungs of mice 12 days after challenge with Bp536 or H762. Anti-CD45–PE antibody was injected i.v. 10 minutes before sacrifice, to specifically label circulating cells (50). Single-cell suspensions of lungs and nose tissues were stimulated with MHC-II peptides and then stained with antibodies to identify IFN-γ– and IL-17–producing Trms. The full gating strategy is shown in Supplemental Figure 3 and determined the percentage and number of CD45^–^CD4^+^CD44^+^CD62L^–^CD69^+^ cytokine-producing Trms.

**Figure 5:**
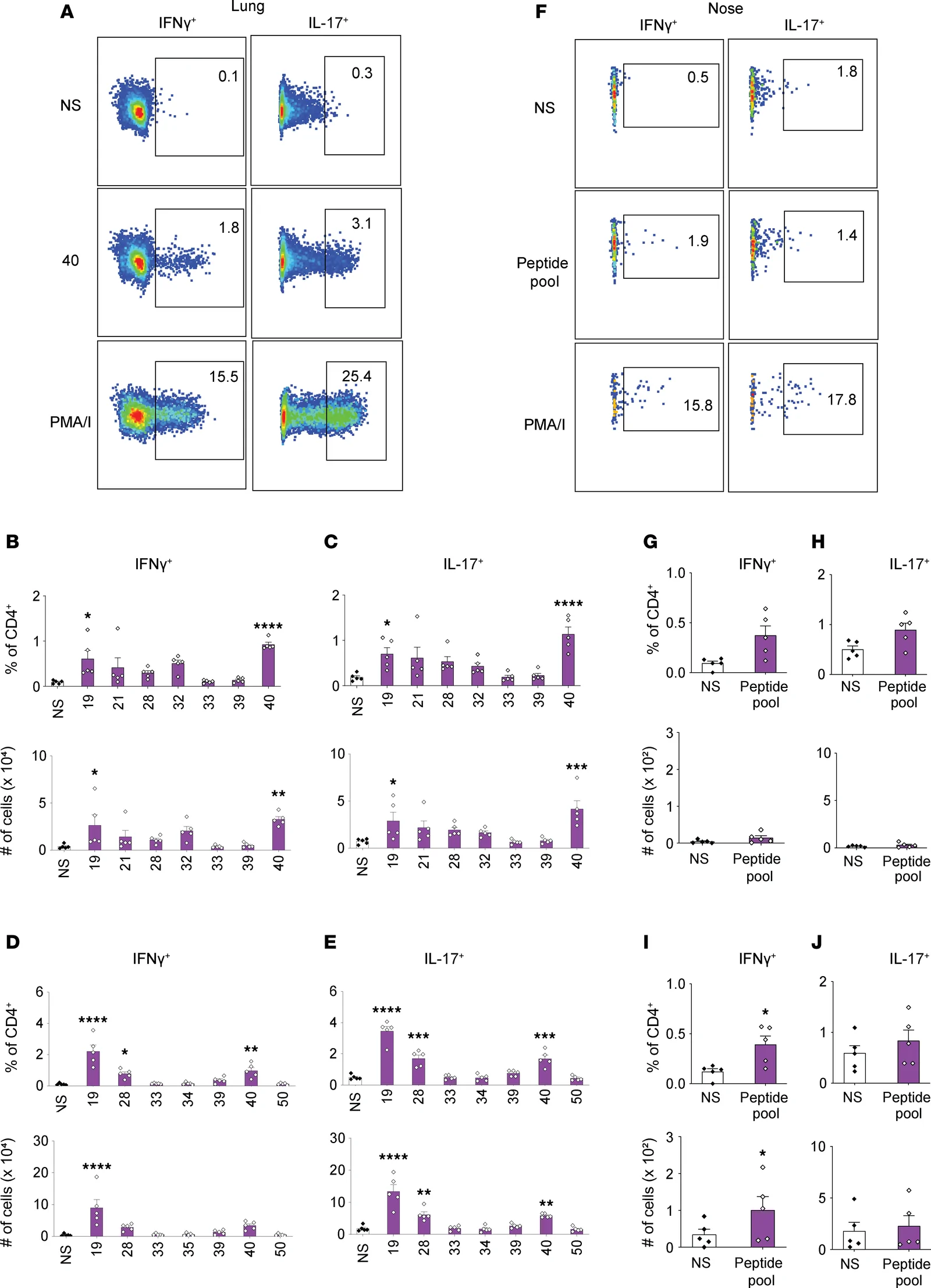
Antigen-specific CD4^+^ Trms are detected in the lungs and nose of *Bp*-challenged mice. (**A**) Representative gating strategy for antigen-specific tissue-resident memory CD4^+^ T cells (Trms) in the lungs of *Bp*-infected mice. NS, nonspecific peptide; peptide 40, representative stimulatory peptide; PMA/I, PMA/ionomycin (positive control). (**B**–**E**) Percentages and numbers of CD45^–^CD4^+^CD44^+^CD62L^–^CD69^+^ cells producing IFN-γ (**B** and **D**) or IL-17 (**C** and **E**) in the lungs of Bp536-infected (**B** and **C**) and H762-infected (**D** and **E**) mice. (**F**) Representative gating strategy for antigen-specific cytokine-producing Trms in nasal tissue. Cells were stimulated with NS peptide, peptide pool (defined in Figure 3), or PMA/I. (**G**–**J**) Percentages and numbers of IFN-γ–producing (**G** and **I**) or IL-17–producing (**H** and **J**) CD45^–^CD4^+^CD44^+^CD62L^–^CD69^+^ cells in the noses of Bp536-infected (**G** and **H**) and H762-infected (**I** and **J**) mice. Data are mean ± SEM; *P < 0.05, **P < 0.01, ***P < 0.001, ****P < 0.0001 compared with NS peptide by ANOVA with Dunnett’s post hoc test. Representative of 2 independent experiments.

Representative flow plots for lungs are shown in Figure 5A. Peptides 19 and 40 stimulated IFN-γ (Figure 5B) and IL-17 (Figure 5C) production by CD4^+^ Trms from Bp536-challenged mice. CD4^+^ Trms from H762-challenged mice produced IFN-γ (Figure 5D) and IL-17 (Figure 5E) following stimulation with peptides 19, 28, and 40. Nose homogenates from individual mice were stimulated with a pool of peptides since the cell yield was insufficient to test the peptides individually. Representative flow plots are shown in Figure 5F. Nose CD4^+^ Trms from Bp536-challenged mice did not produce significant IFN-γ (Figure 5G) or IL-17 (Figure 5H) in response to stimulation with the peptide pool. In contrast, nose CD4^+^ Trms from H762-challenged mice produced IFN-γ (Figure 5I) but not IL-17 (Figure 5J). Thus, CD4^+^ Trms in the respiratory tract elicited by *Bp* infection are antigen specific and recognize a subset of the antigens recognized by systemic T cells.

### PBMCs from wPV-immunized humans recognize peptides from BrkA, YiaO, Cbf2, BP0205, and BP0840.

**Figure 6:**
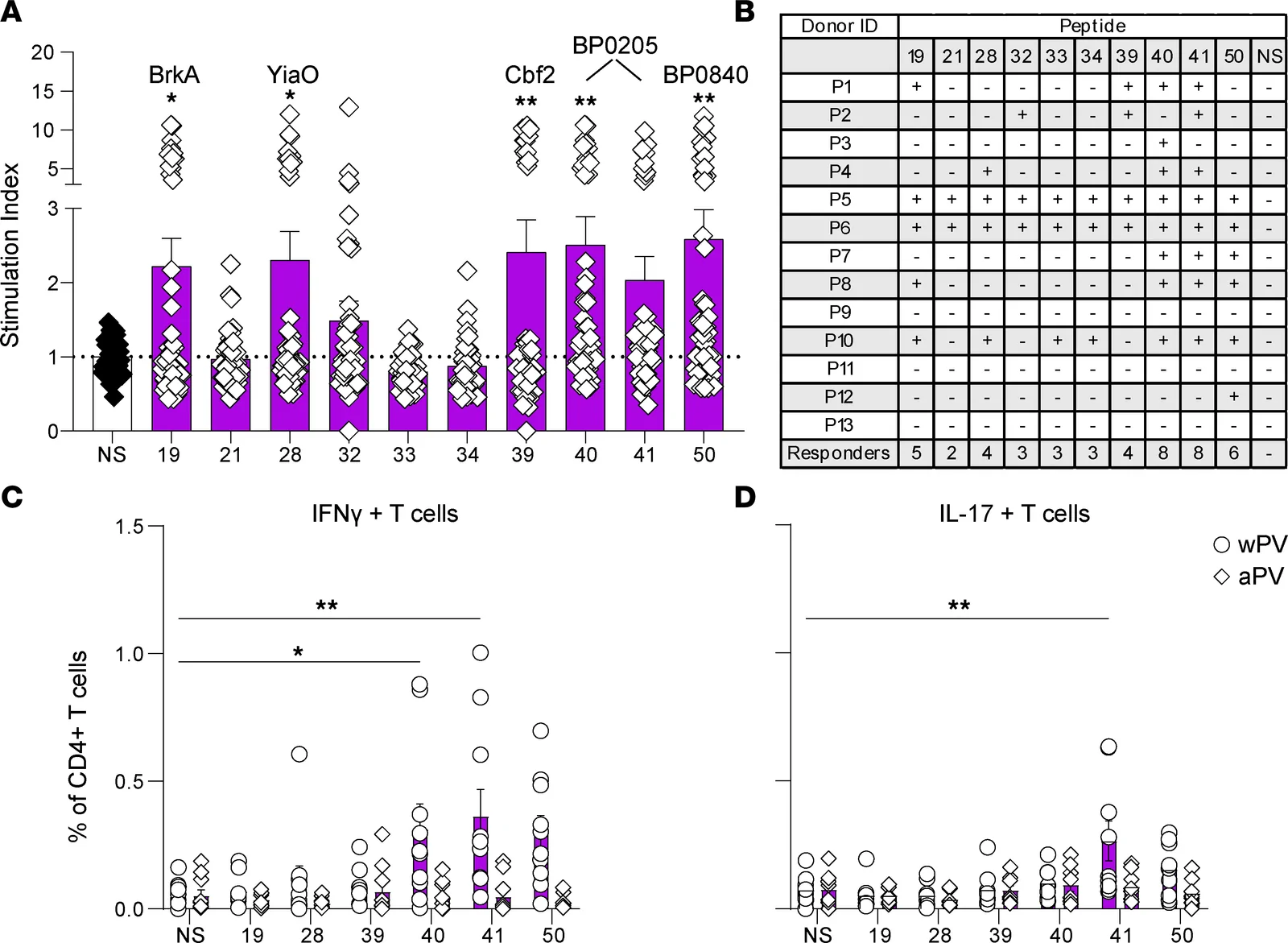
Immunogenic peptides stimulate proliferation and cytokine production of PBMCs from wPV-immunized adults. PBMCs from male (*N* = 7) and female (*N* = 6) adults 27–40 years of age who received wPV immunization as children. PBMCs were stimulated with selected peptides that induced proliferation and cytokine production in mice. (**A**) Stimulation index of individual peptides. (**B**) Proliferation (+) or no proliferation (–) of donor PBMCs in response to stimulation with individual peptides. The data were pooled from 3 independent experiments with at least 3 technical replicates for each peptide for each donor. Mean ± SEM comparing the stimulation index of each peptide versus the NS control peptide. *P < 0.05, **P < 0.01. (**C** and **D**) IFN-γ (**C**) and IL-17 (**D**) production by T cells of PBMCs from wPV-immunized (*N* = 10, 5 male and 5 female) and aPV-immunized (*N* = 10, 5 male and 5 female) donors. Mean ± SEM comparing the stimulation index of each peptide versus the NS control peptide. *P < 0.05, **P < 0.01 by unpaired 2-tailed *t* test.

We tested the hypothesis that CD4^+^ T cells from humans immunized with wPV would recognize the *Bp* antigens identified by screening of the *Bp* immunopeptidome presented on murine MHC-II. PBMCs were isolated from men and women who would have received the wPV as children. PBMCs were stimulated with the peptides that activated murine T cells. The PBMCs were stimulated with selected individual peptides followed by a ^3^H-thymidine pulse to detect proliferating cells. Peptides 19 (BrkA), 28 (YiaO), 39 (Cbf2), 40 (BP0205), and 50 (BP0840) stimulated significant PBMC proliferation (Figure 6A). Peptide 41, an additional high-affinity peptide derived from BP0205 (that did not stimulate murine T cells), also stimulated human PBMCs, although the increase was not significant (*P* = 0.1). Figure 6B shows the responses of individual donors. Each peptide elicited responses from 2–8 donors. Two of the 13 donors (15%) responded to all 10 peptides, and 8 of 13 donors (61%) responded to peptide 40 and peptide 41, suggesting broad recognition of these proteins in humans.

We tested production of IFN-γ and IL-17 by intracellular cytokine staining and flow cytometry as a measure of full effector function. CD4^+^ T cells from wPV-immunized donors stimulated with peptides derived from BP0205 elicited IFN-γ (Figure 6C). Though not significantly, CD4^+^ T cells from some of the wPV donors produced IFN-γ when stimulated with YiaO- and BP0840-derived peptides. Only stimulation with one BP0205 peptide (peptide 41) induced IL-17 expression from CD4^+^ T cells of wPV-immunized donors (Figure 6D). Thus, CD4^+^ T cells of wPV-immunized individuals recognize *Bp* proteins that were not previously considered as vaccine targets. A previous study showed that a subset of T cell antigens recognized by wPV-immunized donors were also recognized by aPV-immunized donors (51) and suggested that these individuals may have had a prior *Bp* infection. We tested aPV-immunized donors and did not detect cytokine production from the PBMCs of aPV-immunized donors (Figure 6, C and D). Thus, prior *Bp* exposure is unclear in these individuals.

### An acellular vaccine formulation containing T cell antigens reduces Bp bacterial burden in the mouse respiratory tract.

**Figure 7:**
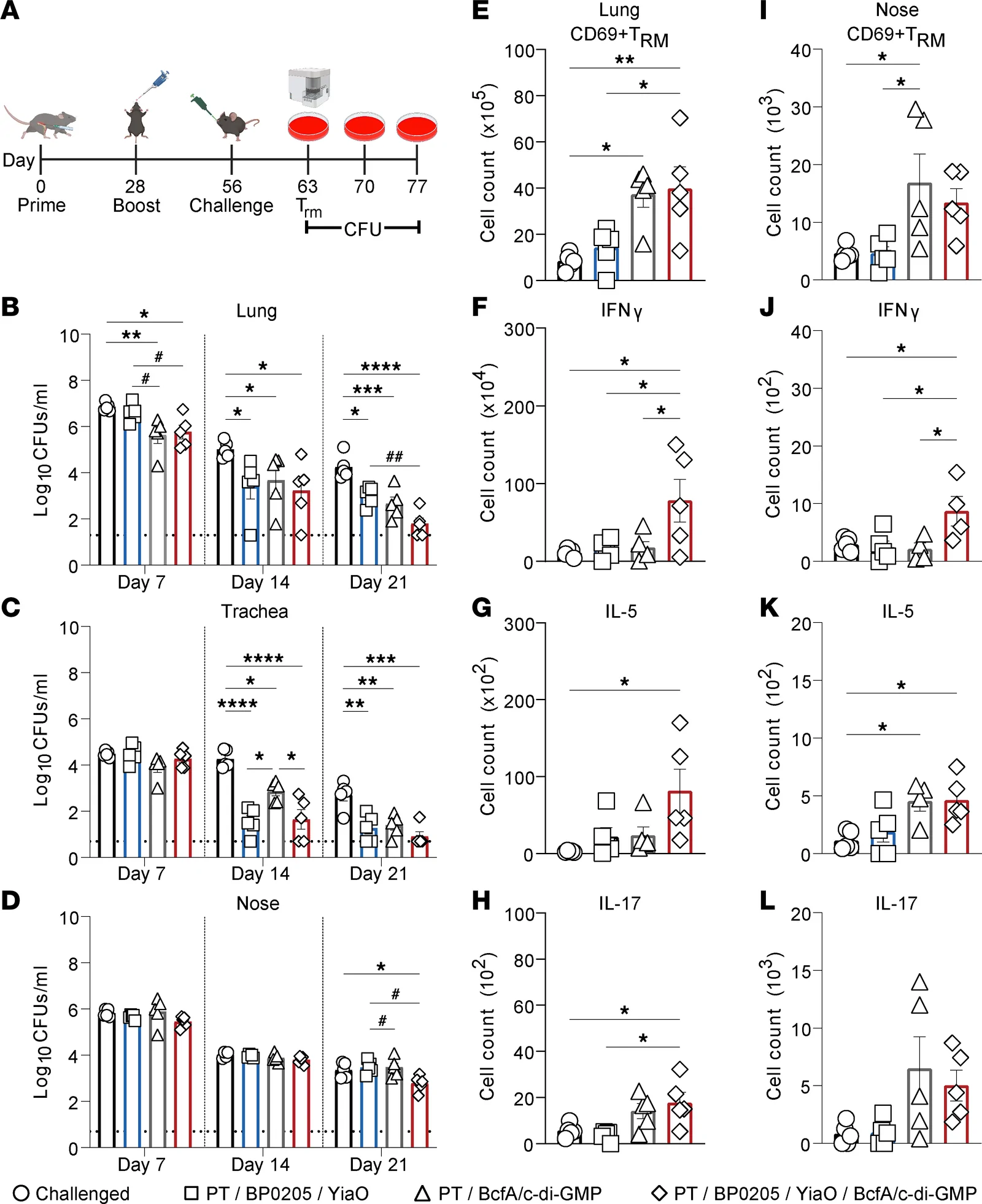
Prime-pull immunization with a subunit vaccine containing BP0205 and YiaO reduces *Bp* respiratory tract colonization. (**A**) Immunization and challenge scheme. (**B**–**D**) Bacterial burden in the lungs (**B**), trachea (**C**), and nose (**D**) was determined on days 7, 14, and 21 after challenge. Dotted line indicates the limit of detection. (**E**–**H**) The number of CD4^+^CD45^–^CD44^+^CD62L^–^CD69^+^ Trms in the lungs (**E**) that express IFN-γ (**F**), IL-5 (**G**), and IL-17 (**H**). (**I**–**L**) The number of CD4^+^CD45^–^CD44^+^CD62L^–^CD69^+^ Trms in the nose (**I**) that produce IFN-γ (**J**), IL-5 (**K**), and IL-17 (**L**). Data shown are mean ± SEM. *P < 0.05, **P < 0.01, ***P < 0.001, ****P < 0.0001 compared with unimmunized mice, and ^#^*P* < 0.05, ^##^*P* < 0.01 comparing immunization groups at each day after challenge, by 1-way ANOVA with Dunnett’s post hoc test. One experiment of 2 independent experiments.

We tested protective capacity of 2 new antigens in an acellular subunit vaccine. YiaO and BP0205 were selected because they were recognized by Bp536- and H762-immune murine systemic and mucosal CD4^+^ T cells and PBMCs of wPV-immunized people. Pertussis toxin (PT) was included since anti-PT antibodies are important for ameliorating the hallmark disease symptoms of pertussis (52, 53). The complete open reading frame, minus the signal sequence, of YiaO and BP0205 was overproduced in BL21DE3 ClearColi bacteria that express a mutant non-signaling LPS (54), and the 6xHis-tagged proteins were purified on a nickel–nitrilotriacetic acid affinity column. Intact proteins of the expected molecular weight were produced (Supplemental Figure 4). A single species was detected for BP0205, while putative trimers of YiaO were also detected. The recombinant proteins were recognized by serum from *Bp*-convalescent mice (Supplemental Figure 5), suggesting that the tertiary structure of the recombinant proteins resembled the endogenous proteins. Mice were immunized with the purified recombinant proteins alone or combined with the STING agonist c-di-GMP (55) and *Bordetella* colonization factor A (BcfA) (56–59) as the adjuvants (Figure 7A), and challenged with Bp536 at 4 weeks after booster immunization with lungs, trachea, and nasal tissue harvested at the indicated time points (Figure 7A). Bacterial burden was reduced in the lungs on day 7 in mice immunized with PT plus adjuvants and PT/BP0205/YiaO plus adjuvants compared with unimmunized challenged mice and mice immunized with antigens alone (Figure 7B). *Bp* burden in the lungs (Figure 7B) was reduced on days 14 and 21 after challenge in all immunized mice compared with naive challenged mice. Mice immunized with PT/BP0205/YiaO plus adjuvants had lower *Bp* burden (near the limit of detection) at day 21 after challenge compared with mice immunized with antigens alone. All vaccine formulations reduced *Bp* burden in the trachea (Figure 7C) at days 14 and 21 after challenge in comparison with naive immunized challenged mice. Bacterial burden in the nose was only reduced in mice immunized with antigens plus adjuvants (Figure 7D) compared with unimmunized mice and mice immunized with antigens alone. Thus, the adjuvanted vaccine formulation reduced bacterial burden in the upper respiratory tract and reduced bacterial burden to the limit of detection in the lower respiratory tract.

We then compared the protection provided by our experimental vaccine with the approved aPV vaccine, Boostrix (GalaxoSmith Kline) Mice were primed i.m. with one-hundredth of the human dose of aPV or PT/BP0205/YiaO/BcfA/c-di-GMP and boosted intranasally (i.n.) on day 28 with the same formulation. Immunized and unimmunized control mice were challenged i.n. with Bp536, and CFUs in the lung and nose were evaluated on days 7, 14, and 21 after challenge. *Bp* colonization of the lungs was reduced in mice immunized with the experimental vaccine on day 7 (Supplemental Figure 6A), day 14 (Supplemental Figure 6B), and day 21 (Supplemental Figure 6C). In contrast, lung colonization in aPV-immunized mice was only reduced by day 21 (Supplemental Figure 6C). Thus, the kinetics of bacterial clearance from the lungs was accelerated in mice immunized with the experimental vaccine compared with aPV. Nasal colonization was reduced by approximately 0.5 log on day 21 in mice immunized with PT/BP0205/YiaO/BcfA/c-di-GMP (Supplemental Figure 6F), while nasal colonization was not reduced in aPV-immunized mice. These data corroborate previous data from our laboratory (59) and others (5, 10, 11) showing that aPV immunization does not reduce bacterial colonization of the upper respiratory tract.

### Antigen-specific CD4^+^ Trms are detected in the respiratory tract following immunization and challenge.

We showed previously that i.m./i.n. immunization with aPV elicits very few CD4^+^ Trms (59) that produce IL-5 but not IFN-γ or IL-17. To test whether our experimental vaccine generated antigen-specific CD4^+^ Trms, we immunized mice with PT/BP0205/YiaO/BcfA/c-di-GMP. We evaluated CD4^+^ Trms on day 7 after challenge with Bp536. The gating strategy used was the same as shown in Supplemental Figure 3. On day 7, the number of CD69^+^ Trms increased in the lungs of mice immunized with PT plus adjuvants and antigens plus adjuvants (Figure 7E) compared with unimmunized challenged mice. The number of IFN-γ^+^ (Figure 7F), IL-5^+^ (Figure 7G), and IL-17^+^ Trms (Figure 7H) increased in the lungs of mice immunized with antigens plus adjuvants compared with naive and unimmunized challenged mice.

While the increase in CD69^+^ Trms was less pronounced in the nose (Figure 7I), there was a significant increase in the number of IFN-γ^+^ (Figure 7J) and IL-5^+^ (Figure 7K) Trms in comparison with unimmunized challenged mice. IL-17^+^ Trms (Figure 7L) in the nasal cavity did not significantly increase.

Mice immunized with antigens alone and antigens plus adjuvants generated significant BP0205-specific antibody titers compared with unimmunized challenged mice (Supplemental Figure 7A). Mice immunized with antigens plus adjuvants and then challenged with *Bp* generated BP0205-specific IgG in the lungs (Supplemental Figure 7B) and nasal cavity (Supplemental Figure 7C) compared with unimmunized challenged mice. Anti-YiaO specific IgG antibodies were detected in the serum of mice immunized with antigens plus adjuvants (Supplemental Figure 7D) but were not detected in the lungs (Supplemental Figure 7E) or nose (Supplemental Figure 7F) following challenge. IgA antibodies were not detected (data not shown). These data show that immunization of mice with antigens identified by our screen generated cellular and humoral immunity.

### Depletion of CD4^+^ T cells prior to challenge abolished protection.

**Figure 8:**
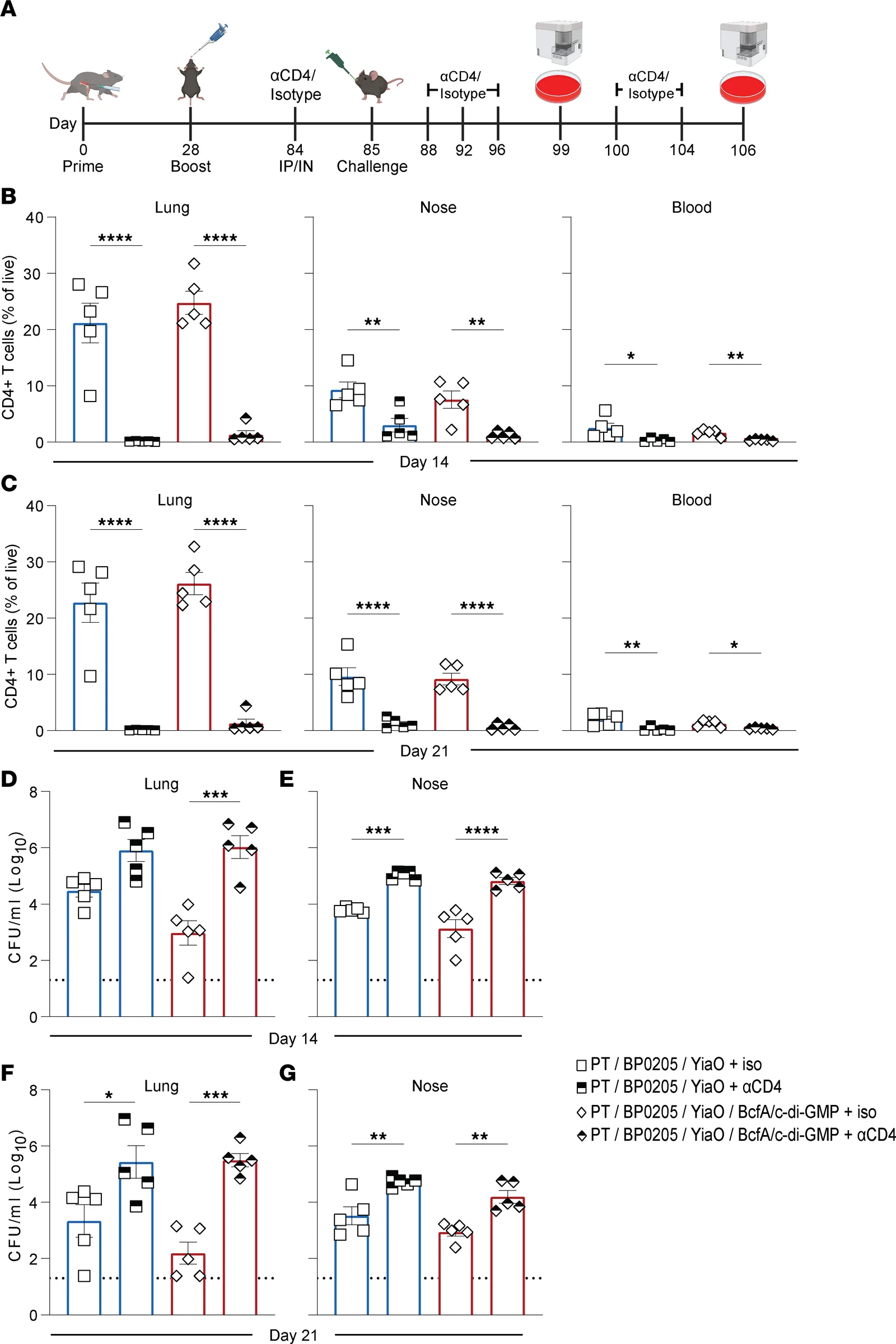
CD4^+^ T cell depletion prevents reduction of bacterial burden in PT/BP0205/YiaO/BcfA/c-di-GMP–immunized mice. (**A**) Experimental outline for immunization, CD4^+^ T cell depletion, bacterial challenge, and analysis. (**B**) T cells in the lungs, nose, and blood on day 14 after challenge. (**C**) T cells in the lungs, nose, and blood on day 21 after challenge. (**D**–**G**) Bacterial burden was enumerated in the lungs (**D**) and nose (**E**) on day 14 after challenge, and in the lungs (**F**) and nose (**G**) on day 21 after challenge. Dotted line indicates the limit of detection. Data shown are mean ± SEM. *P < 0.05, **P < 0.01, ***P < 0.001, ****P< 0.0001 compared with unimmunized mice by 1-way ANOVA with Dunnett’s post hoc test. One experiment of 2 independent experiments.

We then assessed whether CD4^+^ T cell depletion from the mucosa and circulation would prevent reduction of *Bp* from the respiratory tissues in mice immunized with the antigens. Immunized mice were treated with anti-CD4 antibody or isotype control i.p. to deplete circulating T cells and i.n. to deplete mucosal T cells, and challenged with Bp536 on day 0 (Figure 8A). Depletion of CD4^+^ T cells in the peripheral blood, lungs, and nose was confirmed by flow cytometry and showed that CD4^+^ T cells were depleted in the lungs, nose, and blood on day 14 (Figure 8B) and day 21 (Figure 8C). Bacterial CFUs were reduced in the lungs of isotype control–treated mice immunized with PT/BP0205/YiaO plus adjuvants, while CD4^+^ T cell depletion prevented reduction of bacterial burden in the lungs at day 14 (Figure 8D) and day 21 (Figure 8E). *Bp* burden in the nose increased in immunized mice depleted of CD4^+^ T cells on days 14 (Figure 8F) and 21 (Figure 8G) after challenge compared with isotype-treated immunized mice. Serum anti-PT, anti-BP0205, and anti-YiaO antibodies were detected in isotype control– and anti-CD4–treated mice (data not shown), suggesting that antibodies alone were not sufficient to reduce the bacterial burden from the respiratory tract. Thus, CD4^+^ T cells generated by immunization with the BP0205- and YiaO-containing vaccine are required for elimination of *Bp* from the respiratory tract.

## Discussion

In the present work, we undertook an untargeted, discovery mass spectrometry–based immunopeptidomics approach to identify MHC-II binding peptides from Bp536, the *Bp* reference strain, and H762, a recent clinical isolate. Bp536 (a derivative of Tohama I) was isolated in the 1950s from a pertussis patient in Japan. H762 is fully sequenced and was isolated from a patient during a pertussis outbreak in the United States in 2011. It lacks expression of pertactin, one of the aPV antigens, and represents one of the predominant *Bp* clades currently circulating in the United States (60). Circulating clinical *Bp* strains exhibit considerable genetic diversity compared with the reference strain, resulting in varying expression of *Bp* virulence factors and aPV antigens (12, 61).

We reasoned that a direct immunopeptidomics analysis of APCs treated with bacterial lysate, as described previously for *Listeria monocytogenes* (30), would identify the antigens that are presented in vivo following immunization or infection. The depth and complexity of the MHC-II–presented *Bp* immunopeptidome have not been reported. The MHC-II immunopeptidomes contained more than 340–564 peptides presented on MHC-II that were derived from 154–211 *Bp* proteins, and suggested that many putative antigens may be recognized by T cells responding to *Bp*.

Because peptides with high affinity for MHC-II are more likely to elicit CD4^+^ T cell responses in vivo (34, 62–64), we leveraged a bioinformatics approach to prioritize a 50-peptide set with predicted high affinity for murine MHC-II I-A^b^, which we used for functional testing. Surprisingly, only a small number of epitopes stimulated proliferation and a smaller subset of these epitopes elicited cytokine production from murine immune CD4^+^ T cells. Thus, all high-affinity epitopes that are displayed on MHC-II molecules with similar abundance are not equally immunogenic in vivo. Two epitopes (peptides 30 and 31) from the aPV antigen zzzfilamentous hemagglutinin (FHA) with predicted high affinity for I-A^b^ were identified in the Bp536 IPs. However, these peptides did not activate Bp536- or H762-immune effector CD4^+^ T cells. Epitopes from other aPV antigens were not identified through this analysis, suggesting that aPV antigens may not be immunodominant T cell determinants, offering a potential explanation for the weak and short-lived immunity elicited by aPV immunization (65–67).

Several proteins recognized by Bp536- and H762-immune CD4^+^ T cells were conserved in other recent clinical isolates (20), suggesting that these MHC-II antigens may elicit broadly cross-protective immune responses. However, some epitopes were only recognized by Bp536- or H762-immune T cells, suggesting that the immunodominant antigens in the *Bp* reference and clinical isolates are variable. In ongoing work, we are testing the epitopes unique to H762 to pinpoint additional antigens that may be important for protection against current *Bp* isolates.

A recent study (51) mapped human CD4^+^ T cell responses to the *Bp* proteome by combining large-scale epitope prediction with experimental validation using PBMCs from a diverse human cohort. This approach identified several antigens that were recognized by human PBMCs from wPV- and aPV-immunized individuals, suggesting that some aPV-immunized people in their cohort may have had a prior *Bp* infection. This analysis also showed that aPV antigens account for a limited fraction of the T cell response to *Bp*. Most of the response was directed to intracellular and extracellular proteins with functions in virulence, metabolism, and protein folding. The source proteins of the immunogenic epitopes we identified also have varied cellular functions, including inhibition of the complement pathway (BrkA) (68), solute transport (YiaO) (69), and protein export (SecD, also known as BP0205) (70). Thus, CD4^+^ T cells responding to *Bp* infection recognize intracellular and extracellular proteins with potential pathogenic or physiological functions in the *Bp* life cycle.

Another study used a comparative genomics–based approach to predict *Bp* peptides presented on HLA-DR and identified 2 peptides that were recognized by CD4^+^ T cells of wPV-immunized individuals (71). However, it is not clear that the epitopes identified in either study generate T cell responses upon vaccination or natural infection.

Only one of the source proteins (BrkA) identified by our study was also identified in a previous study (51). These differences highlight the importance of our holistic approach that combines immunopeptidomics of cells treated with bacterial lysates, MHC prediction algorithms, and functional T cell assays to identify the best vaccine candidates. In addition, variations in the study cohorts used to screen the antigens may also influence identification of immunogenic epitopes.

Importantly, several peptides that were recognized by murine CD4^+^ T cells were also recognized by PBMCs from wPV-immunized men and women, suggesting that these antigens could elicit protective immunity in humans. Our cohort of aPV-primed donors did not respond to the antigens, suggesting that they may not have been exposed to a *Bp* infection.

As a proof of concept, we tested the ability of an acellular vaccine formulation containing BP0205 and YiaO to protect mice against a *Bp* challenge. We showed that BcfA activates immune responses through TLR4 (58) and mice immunized with a BcfA-adjuvanted vaccine generate Th1/Th17 responses in the nose and the lungs (59). Another group reported Th1/Th17 responses generated by the STING agonist c-di-GMP (72). We reasoned that combining these two adjuvants would elicit a strong Th1/Th17 response. While our vaccine elicited this phenotype, the Th1 responses were predominant. *Bp* bacterial burden was reduced to the limit of detection in the lungs and trachea and significantly reduced in the nose of immunized mice, demonstrating the protective capacity of a vaccine containing T cell–recognized antigens previously untested in a subunit vaccine. As ongoing screening identifies other antigens that meet our criteria of recognition by murine and human T cells, we will test their ability to protect mice using other adjuvant combinations that drive strong Th17 responses, especially in the upper respiratory tract.

We demonstrated the critical role of CD4^+^ T cells generated through immunization of mice with the new vaccine, by depleting systemic and mucosal CD4^+^ T cells before challenge. Reduction of bacterial burden in the upper and lower respiratory tract was prevented in mice immunized with antigens plus adjuvants, clearly showing that CD4^+^ T cells elicited by the vaccine are necessary for protection. To our knowledge, these antigens have not been tested previously in a subunit vaccine. PT is included in all pertussis vaccines, since anti-PT antibodies are important for preventing disease (73, 74). Anti-BP0205 and anti-YiaO antibodies, while present, are unlikely to directly eliminate intact *Bp* from the respiratory tract since they recognize intracellular proteins.

Our study has some limitations. (a) To identify potential antigens, we considered the 3 critical factors of peptide abundance, MHC-II affinity, and T cell avidity, but other factors, such as stability on MHC, were not included. The immunopeptidome included several hundred peptides with predicted low affinity for I-A^b^. We will test additional epitopes in subsequent work. (b) Tissue-resident T cell responses are critical for sustained protection against infection in the mucosa (5, 17, 48, 49). Nasal CD4^+^ Trm responses, though significant, were not sufficient to reduce the bacterial burden to the limit of detection. This may be due to the weak Th17 responses elicited in the nose. We are testing different antigen/adjuvant combinations to optimize the Th17 response and hypothesize that increased Th17 responses may effectively reduce nasal colonization. These may be induced by a second i.n. booster to enhance nasal CD4^+^ T cell responses (75). Optimization of the adjuvant combination to increase nasal Th17 responses may also enhance protection. (c) Antigens recognized by nasal T cells may differ from those recognized by systemic and lung T cells. We are testing the immunopeptidome for antigens that are preferentially recognized by nasal CD4^+^ Trms. (d) As the vaccine formulation is optimized, key manufacturing and distribution considerations, such as protein stability and scalability, will be evaluated to support clinical translation.

In summary, we show that combining immunopeptidomics and functional T cell assays is a high-resolution approach to identify immunodominant CD4^+^ T cell–recognized targets that elicit protective immunity. Incorporation of these antigens in next-generation vaccines for infants and adults may better control *Bp* resurgence and reduce the public health burden of pertussis.

## Methods

### Sex as a biological variable.

All experiments were conducted with samples from male and female mice and humans. The data are presented in aggregated form.

### Bacterial strains, media, and growth conditions.

*Bp* strains Bp536 and H762 were maintained on Bordet-Gengou (BG) agar (Difco) plates containing 10% defibrinated sheep’s blood (HemoStat) supplemented with 100 μg/mL streptomycin or 20 μg/mL cephalexin, respectively. Liquid bacterial cultures were grown overnight at 37°C on a roller drum to OD_600_ ≈ 1.0 in Stainer-Scholte medium plus 1 mg/mL dimethyl-β-cyclodextrin (Acros Organics). Bacteria were heat-inactivated (65°C, 30 minutes) and diluted in BMDC growth medium for stimulation.

For immunizations, heat-inactivated bacteria were diluted in saline to 1 × 10^9^ CFU/mL. For mouse challenges, live bacteria were diluted to 1 × 10^7^ CFU/mL.

### BMDC differentiation.

BMDCs were prepared as previously described (27). Briefly, bone marrow was isolated from C57BL/6J mice and dissociated, and red blood cells were lysed with ACK lysis buffer (Thermo Fisher Scientific, Gibco A10492), resuspended in T cell medium (RPMI 1640 plus 10% FBS [MilliporeSigma, F2442], 10 μg/mL gentamicin [Gibco, 15710064], 5 × 10^–5^ M β-mercaptoethanol [Thermo Fisher Scientific, 125472500], 40 ng/mL GM-CSF [BioLegend, 576306]), and seeded in 10 cm^2^ non-tissue-culture-treated Petri dishes (VWR, 25384-342) at 5 × 10^6^ to 10 × 10^6^ cells per plate. Half of the medium was replaced every 2 days. On day 6–7 after differentiation, BMDCs were transferred to 10 cm^2^ tissue culture–treated plates (Fisher Scientific, Falcon 353046) at 1 × 10^7^ cells per plate, and the next day, HKBp was added at an MOI of 100:1 for 24 hours. Cells were harvested by scraping and snap-frozen until IP.

### Flow cytometry analysis.

All antibodies used for flow cytometry are listed in Supplemental Table 3.

BMDCs were stained with antibodies against CD11c, MHC-II I-A^b^, CD40, CD80, and CD86, collected on a Cytek Aurora spectral flow cytometer, and analyzed using FlowJo software version 10.8.0.

### Immunoaffinity purification of MHC-II complexes.

To immunoprecipitate MHC-II I-A^b^, frozen cell pellets were lysed in cold IP lysis buffer (1.5% Triton X-100 [MilliporeSigma, T9284], 100 mM NaCl [MilliporeSigma, 71386], 6 mM MgCl_2_ [MilliporeSigma, 63069], 20 mM Tris [pH 8; Invitrogen, 15568025], 1 mM EDTA [Invitrogen, 15575-038], 60 mM octylglucoside [MilliporeSigma, O8001], 0.2 mM iodoacetamide [MilliporeSigma, I1149], 4 U/mL Benzonase [MilliporeSigma, E1014-25KU], 1X protease inhibitor cocktail [MilliporeSigma, 11873580001], 1 mM PMSF [MilliporeSigma, 93482]) at 5 × 10^7^ cells per mL lysis buffer per sample.

Clarified lysates were immunoprecipitated overnight at 4°C with gentle end-over-end rotation using 250 µL of packed GammaBind Plus Sepharose beads (MilliporeSigma, GE17-0886-01) covalently coupled to 70 µg of purified anti-H2-IAb antibody (clone Y3P, Bio X Cell). Beads were then washed 4 times in a wash buffer containing 60 mM octylglucoside, 0.2 mM iodoacetamide, followed by 4 washes with 10 mM Tris (pH 8.0) and a final wash with HPLC-MS–grade water (J.T.Baker, Avantor, JT9831-3). Finally, samples were freeze-dried and stored at –80°C until peptide isolation and mass spectrometry (30).

### MHC-II peptide elution and desalting.

MHC-II peptide elution was performed as previously described (76), except that a Sep-Pak tC18 cartridge (Waters, WAT054960) replaced the Sep-Pak plate. Beads were resuspended in 3% acetonitrile (ACN)/5% formic acid (FA) and transferred to the equilibrated cartridge without a filter plate. After elution, peptides were frozen at –80°C and lyophilized.

Peptides were reconstituted in 10 mM Tris-HCl (pH 7.5), reduced with 5 mM dithiothreitol (DTT; Thermo Fisher Scientific, A39255) for 20 minutes at 50°C with shaking, then alkylated with 16.25 mM iodoacetamide (Thermo Fisher Scientific, A39271) for 30 minutes (room temperature) while shaking in the dark. The reaction was quenched with 5 mM DTT for 15 minutes at room temperature while shaking. The sample was brought to 3% ACN/5% FA by addition of 90 μL of 6.67% ACN/11.1% FA.

A secondary desalting of the reduced and alkylated samples was performed by micro-scaled basic reversed-phase separation on SDB-XC stage tips as previously described (76). Peptides were frozen at –80°C, lyophilized, and stored at –80°C (77). 

### LC-MS/MS analysis.

MHC-II I-A^b^ immunopeptidome data collection by LC-MS/MS was performed as described previously for HLA-DR (77). Lyophilized peptides were resuspended in 3% ACN/5% FA and injected onto an analytical C18 column (25–30 cm, 1.9 μm ReproSil-Pur C18 silica beads [Dr. Maisch HPLC GmbH], packed in-house PicoFrit 75 μm diameter, 10 μm emitter [New Objective, PF360-75-10-N-5]) connected to an Orbitrap Exploris 480 (Thermo Fisher Scientific) with a Vanquish Neo (Thermo Fisher Scientific). Peptides were eluted by a linear gradient (solvent A, 0.1% FA in 99.9% water; solvent B, 0.1% FA in 99.9% ACN) from 6% to 30% solvent B over 84 minutes, 30% to 90% solvent B over 9 minutes, and finally held at 90% solvent B for 5 minutes at 200 nL/min. On the Exploris 480, MS1 spectra were collected until either 100% normalized automatic gain control (AGC) or a maximum injection time of 50 milliseconds was reached, within a scan range of 350–1,700 *m*/*z* at 60,000 resolution. Monoisotopic peak determination was set as “peptide” with relaxed restrictions when too few precursors were found. Precursor fit window was 1.4 *m*/*z* with a threshold of 50%. Dynamic exclusion was 10 seconds at 10 ppm. Precursors of charges 2 to 5+ were subjected to MS/MS acquisition. Potential precursors above 1.0 × 10^3^ intensity were isolated in a 1.1 *m*/*z* window and collected until either a 50% normalized AGC target or 100-millisecond maximum injection time was reached. Precursors were fragmented with 34% high-energy C-trap dissociation (HCD) collision energy at 15,000 resolution. One H762 peptide replicate was desalted twice and injected without reduction and alkylation; the subsequent sample was reduced, alkylated, desalted, and re-injected.

### MHC-II peptide database search.

Raw mass spectra were interpreted with the Spectrum Mill (SM) software package, version 8.02 (Broad Institute; proteomics.broadinstitute.org). Only MS/MS spectra with a precursor sequence MH+ of 600–4,000 Da, a precursor charge less than 5+, and a minimum of less than 5 detected peaks were searched. Similar spectra with the same precursor *m*/*z* acquired in the same chromatographic peak were not merged. Carbamidomethylation of cysteine was set as a fixed modification in the SM Extraction module. Before searching, all MS/MS spectra had to pass the spectral quality filter with a sequence tag length greater than 1 (i.e., minimum of 3 peaks separated by the in-chain masses of 2 consecutive amino acids).

MS/MS spectra were searched against a database of a GENCODE vM31 mouse reference proteome with 36,888 entries, 642 common laboratory contaminants, and the reference proteome of either the H762 strain (UP000255014) or the Bp536 strain (UP000002676) (i.e., Tohama I proteome). Parameters for SM MS/MS MHC-II search module included: no enzyme specificity; ESI-QEXACTIVE- HCD-HLA-v3 30 scoring; fixed modifications: carbamidomethylation of cysteine; variable modifications: protein N-terminal acetylation, oxidized methionine, pyroglutamic acid at peptide N-terminal glutamine, deamidation NG of asparagine; precursor mass shift range of –18 to 33 Da; precursor mass tolerance of ±10 ppm; product mass tolerance of ±10 ppm; and minimum matched peak intensity of 30%.

Peptide spectrum matches (PSMs) within less than 1% false discovery rate were confidently assigned for individual spectra via the target decoy estimation of the SM Autovalidation module. PSMs were filtered for precursor charges of +2 to +5, sequence lengths of 9–40 amino acids, and a minimum backbone cleavage score (BCS) of 5. BCS is a metric of peptide sequence coverage to enforce uniformly higher minimum sequence coverage for each PSM. The score is a sum after assigning of a 1 or 0 between each pair of adjacent amino acids in the sequence (maximum score is peptide length –1) given all ion types, with the goal of decreasing false-positive spectra having fragmentation in a limited portion of the peptide by multiple ion types.

PSMs were consolidated to peptides through the SM Protein/Peptide Summary module in the case-sensitive peptide-distinct mode. A distinct peptide is determined by the highest-scoring PSM of a peptide detected for each sample. If different modification states were observed for a peptide, each was reported with a lowercase letter indicating a variable modification (e.g., c, cysteinylated; C, carbamidomethylated).The log_10_-transformed peptide intensity (i.e., the proxy for relative abundance) is reported in the SM protein/peptide summary.

Peptides from replicate 2 of the H762 IP were not reduced and alkylated, therefore, a variable modification of cysteinylation of cysteine and precursor mass shift range of –18 to 81 Da was employed in the SM MS/MS MHC-II search module. All other parameters remained the same. Supplemental Table 1 shows the LC-MS/MS–identified peptides for Bp536 and H762.

### GibbsCluster analysis.

GibbsCluster 2.0 was used to obtain 9-mer binding cores from *Bp* and mouse peptides for subsequent sequence logos. The MHC-II ligand parameters were used, with the number of clusters set to 1, and outliers were not removed (78).

### MHC-II binding predictions.

The peptide library was interrogated using the consensus or NetMHCpan algorithms in the Immune Epitope Database (IEDB) (35, 36) for binding to I-A^b^ and at least one human HLA-DR/DQ/DP allele. High-affinity binders, defined as having adjusted percentile rank of ≤10, were selected.

### Peptide library synthesis.

Selected peptides were synthesized to greater than 85% purity by Biosynth and desalted. Stocks were prepared at 20 mg/mL in purified water or DMSO and stored at –20°C.

### E. coli overexpression, purification, and verification of YiaO and BP0205.

The full-length open reading frame of candidate antigens from Tohama I reference strain (GenBank: BX470248.1) was codon-optimized for *E*. *coli* expression, synthesized, and cloned into the 6xHis-tagged expression vector pET-28a (Biomatik Inc.). Integrity was verified by sequencing and restriction mapping. The plasmids were transformed into ClearColi. One-millimolar IPTG (Research Products International, 367-93-1) was added to bacteria at log phase of growth to stimulate protein production for 16 hours. YiaO was isolated from inclusion bodies, and BP0205 was isolated from the soluble fraction and purified on an anti-His affinity column as previously described (79). The purified proteins were dialyzed into aqueous buffer (urea [Research Products International, catalog 57-13-6] [dialyzed 6M–0M], 10 mM Tris [Fisher Scientific, 77-86-1], and 5% glycerol [Fisher Scientific, 56-81-5]) in water at a pH of 8.0. Proteins were stored in 1 mM PMSF (MilliporeSigma, 329-98-6) at –80°C until use. Protein integrity was confirmed by SDS-PAGE (12% gel) and Coomassie blue staining, and protein concentrations determined by Bradford assay (Bio-Rad, 5000006).

The purified proteins were tested for recognition by serum of mice that were infected with *Bp* and had cleared the infection from the respiratory tract at least 2 months earlier (convalescent mice). Briefly, high-binding plates (Corning, reference 9018) were coated with purified proteins (1 μg/mL), blocked with ELISA diluent (Invitrogen, reference 88-7120-88), and then incubated with convalescent serum (1:500 dilution), followed by washing and incubation with goat anti-mouse HRP secondary antibody (Invitrogen, 31430; 1:10,000 dilution). Binding was detected with TMB reagent (BioLegend, 421501) and color change quantified on a plate reader (Bio-Rad).

### Mouse immunizations and challenges.

For heat-killed whole-cell bacteria immunization, mice were lightly anesthetized with 2.5% isoflurane/O_2_ and injected intramuscularly with a dose of 1 × 10^8^ CFU of heat-killed Bp536 or H762 in a 100 μL volume divided between both forelimbs.

For the vaccine protection study, C57BL/6 mice (6 weeks old) were immunized i.m. with genetically detoxified PT, 0.08 μg/animal/dose (BioNet Asia) or together with 1 μg of recombinant proteins BP0205 and YiaO. Mice were immunized with antigens alone or with the adjuvant combination of 10 μg BcfA (56, 58) and 10 μg c-di-GMP (STING agonist; InvivoGen Inc., Vac-nacdg). Vaccine components were mixed and rotated for 30 minutes at room temperature before injection. Mice were boosted i.n. 28 days after priming with the same formulation and vaccine dose. Mice were challenged i.n. with 5 × 10^5^ CFU of *Bp* (strain Bp536) 2 weeks after booster immunization. After euthanasia, lungs and nasal tissue were harvested, homogenized, and plated on BG plates to determine bacterial burden at days 7, 14, and 21 after challenge.

### T cell proliferation assays.

Splenocytes from heat-killed bacteria–immunized mice were collected 1–3 months after booster immunization and pooled, and CD4^+^ T cells were isolated by negative selection (Stem Cell Technologies, EasySep kit 19852). CD4^+^ T cell–depleted splenocytes (Stem Cell Technologies, EasySep kit 18952) from naive mice served as APCs. Purity was confirmed by flow cytometry. For proliferation assays, 6 × 10^4^ CD4^+^ T cells and 1.8 × 10^5^ APCs were cultured in 96-well plates (Corning, 353072) (6 wells per peptide) and stimulated with peptides (10 μg/mL) for 48 hours.

Peripheral blood was collected from wPV-immunized (27–40 years old) and aPV-immunized (18–25 years old) individuals. PBMCs were isolated by Ficoll (MilliporeSigma, 17144003) density gradient and cryopreserved in 90% FBS plus 10% DMSO (MilliporeSigma, D4540). Cells were thawed in T cell medium, plated at 1 × 10^7^ cells/mL, and stimulated with peptides (10 μg/mL) for 72 hours. A nonspecific peptide served as a negative control, and HKBp (MOI 100:1) as a positive control. ^3^H-thymidine (35 μCi/well; PerkinElmer) was added for 18 hours and incorporation measured after harvesting. The stimulation index (SI) was calculated as the ratio of peptide-stimulated to control wells (80).

### T cell functional avidity.

Purified splenic CD4^+^ T cells from HKBp-immunized mice were cocultured with CD4^+^ T cell–depleted naive splenocytes and stimulated with peptides for 72 hours, followed by ^3^H-thymidine incorporation for 12–18 hours. Each peptide was tested in 4 wells.

### Intracellular cytokine staining of human PBMCs.

PBMCs from wPV- and aPV-immunized individuals were plated at 1 × 10^6^ cells per well and stimulated overnight with 2 μg/mL peptides in the presence of protein transport inhibitors. Cells were stained with Zombie NIR viability dye, blocked with 10% FBS, and surface-stained for CD4. After fixation and permeabilization, intracellular IFN-γ and IL-17 were stained. Samples were analyzed on a Cytek Aurora cytometer and processed using FlowJo v11.

### Splenocyte stimulation and cytokine ELISA assays.

Splenocytes were plated at 2.5 × 10^6^ cells per well in 48-well plates with complete T cell medium and stimulated for 72 hours with 1 μg/mL target or control peptides. IFN-γ (R&D Systems, DY485), IL-5 (R&D Systems, DY405), and IL-17 (R&D Systems, DY421) in supernatants were measured by sandwich ELISA per the manufacturer’s instructions. Absorbance was read at 450 nm, and cytokine concentrations were calculated from standard curves.

### Intravascular staining to discriminate between circulating and resident CD4^+^ T cells.

Anti-CD45–PE (3 μg in 100 μL of sterile PBS) was injected i.v. (retro-orbitally) 10 minutes before sacrifice to label circulating lymphocytes. Peripheral blood was collected and checked by flow cytometry to confirm that more than 90% of circulating lymphocytes were CD45-PE^+^.

### Mouse tissue dissociation and flow cytometry analysis.

Mouse lungs were isolated and processed into a single-cell suspension using the gentleMACS tissue dissociator and the mouse Lung Dissociation Kit (Miltenyi Biotec, catalog 130-095-927) and filtered through a 40 μm filter, and RBCs were lysed with ACK (ammonium-chloride-potassium) lysis buffer. The entire nasal septum, including the turbinate and nasal-associated lymphoid tissue, was enzymatically digested using the same kit and filtered, followed by RBC lysis.

Cells from each mouse were resuspended in T cell medium and stimulated with PMA (50 ng/mL)/ionomycin (500 ng/mL) or with 1 μg/mL (0.5 μM) of individual peptides for 5 hours at 37°C with protein transport inhibitor cocktail (eBioscience, 00-4980-93). Samples from mice immunized with BP0205 were stimulated with 1 μg/mL of BP0205 peptide or an I-A^b^–binding negative control peptide for 5 hours at 37°C in the presence of a protein transport inhibitor cocktail.

Cells were washed with cold PBS and stained with Zombie NIR viability dye (BioLegend) for 30 minutes at 4°C. After washing in FACS buffer (PBS plus 1% FBS), cells were Fc-blocked (anti-CD16/CD32) for 5 minutes at 4°C and surface-stained for 20 minutes at 4°C with antibodies against CD3, CD4, CD44, CD62L, and CD69. After washes, cells were fixed (20 minutes, room temperature), permeabilized, and stained intracellularly (30 minutes, 4°C) with IFN-γ, IL-17, and IL-5 antibodies. FMO controls were included. Cells were washed, resuspended in FACS buffer, and acquired on a Cytek Aurora cytometer, then analyzed using FlowJo v10.8.0 (BD Biosciences). Cell counts were calculated by multiplication of population frequency by total live cell numbers.

The purity of CD4^+^ T cells and CD4^+^ T cell–depleted splenocytes was determined by staining of cells before and after enrichment with anti-CD3, anti-CD4, and anti-CD45.

### CD4^+^ T cell depletion.

Immunized mice received 200 μg anti-CD4 depleting antibody or isotype control i.p. and 50 μg i.n. every 4 days starting 1 day before *Bp* challenge. CD4^+^ T cell depletion was confirmed by flow cytometry in lung, nasal tissue, and blood at days 14 and 21 after challenge.

### Statistics.

The mean ± SEM (standard error of the mean) of each group was compared with that of the appropriate control group and significance calculated by ANOVA with Dunnett’s post hoc test. Grubbs’s test identified a single statistical outlier with α = 0.05 that was removed from Figure 2. For pairwise comparisons, an unpaired 2-tailed *t* test was used to determine significance. The data were analyzed and plotted using GraphPad Prism version 9.

### Study approval.

All animal experiments were approved by The Ohio State University Institutional Animal Care and Use Committee under protocol 2017A00000090-R2. Peripheral blood was collected at the Clinical Research Center from human volunteers under The Ohio State University Institutional Review Board protocols 2020H0404 and 2021H0179.

### Data availability.

All data are available in the main article text or in the Supporting Data Values file.

The original mass spectra, peptide spectrum matches, and the protein sequence databases used for searches were deposited in the public proteomics repository MassIVE (https://massive.ucsd.edu) and are accessible at ftp://massive-ftp.ucsd.edu/v10/MSV000098648/.

## Author contributions

PD conceptualized the study. PD, GMH, EKV, and JGA developed methodology. MMS, JMH, GML, GMH, ARA, YL, ALR, JGA, and PD performed investigation. MMS, JMH, GMH, JGA, and PD performed visualization. PD and RD acquired funding. PD, MMS, JMH, RD, GMH, GML, and JGA wrote the manuscript. PD is the senior author on the project and is therefore listed last. JGA is a significant contributor and oversaw the mass spectrometry experiments, and hence shares senior authorship with PD.

## Conflict of interest

JGA is a paid consultant to Moderna.

## Funding support

This work is the result of NIH funding, in whole or in part, and is subject to the NIH Public Access Policy. Through acceptance of this federal funding, the NIH has been given a right to make the work publicly available in PubMed Central.

- NIH/NIAID 1R01AI153829 (to PD and RD).

## Supplementary Material

Supplemental data

Unedited blot and gel images

Supplemental table 1

Supplemental table 2

Supplemental table 3

Supporting data values

## Version 1 09/08/2026

Electronic publication
