# Supplementary material for: Discovery of CD4+ T cell–recognized B. pertussis antigens that reduce airway colonization
Source: JCI Insight. 2026 Sep 8;11(17):e199234. doi: 10.1172/jci.insight.199234 (PMC13564080; doi:10.1172/jci.insight.199234)
Supplement: Supplementary file 1 [file jciinsight-11-199234-s060.pdf]

Supplementary Figures

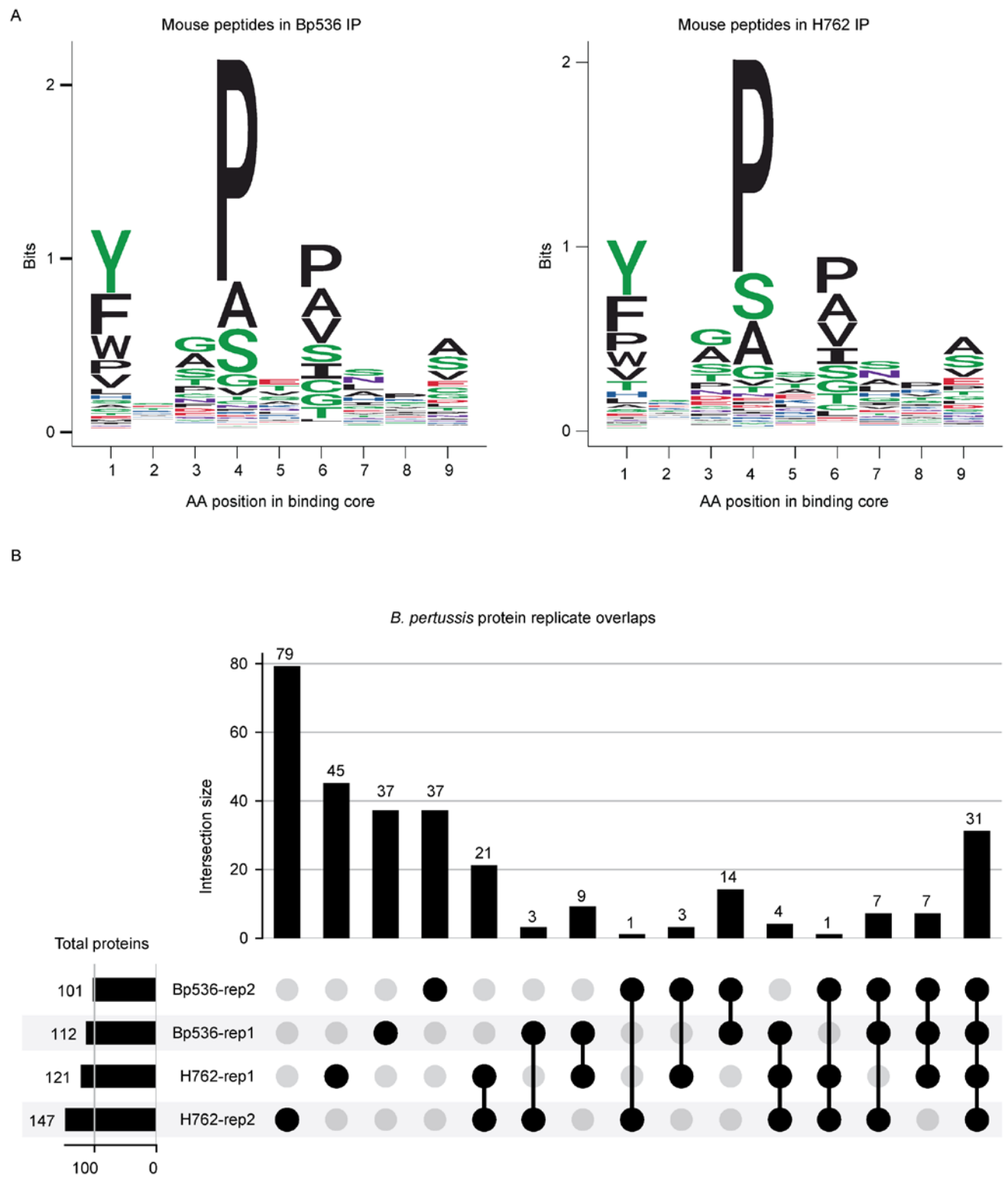

Supplementary Figure 1: Overlap analysis of MHC-II peptide source proteins derived from *Bp* **(A)** UpSet plot displaying the MHC-II peptide source protein overlap between individual Bp536 and H762 IPs and across the two strains. The overlap of source proteins between strains was determined based on full length protein sequences. **(B)** Motifs of mouse peptides immunoprecipitated from I-A^b of Bp536 and H762 treated BMDCs.

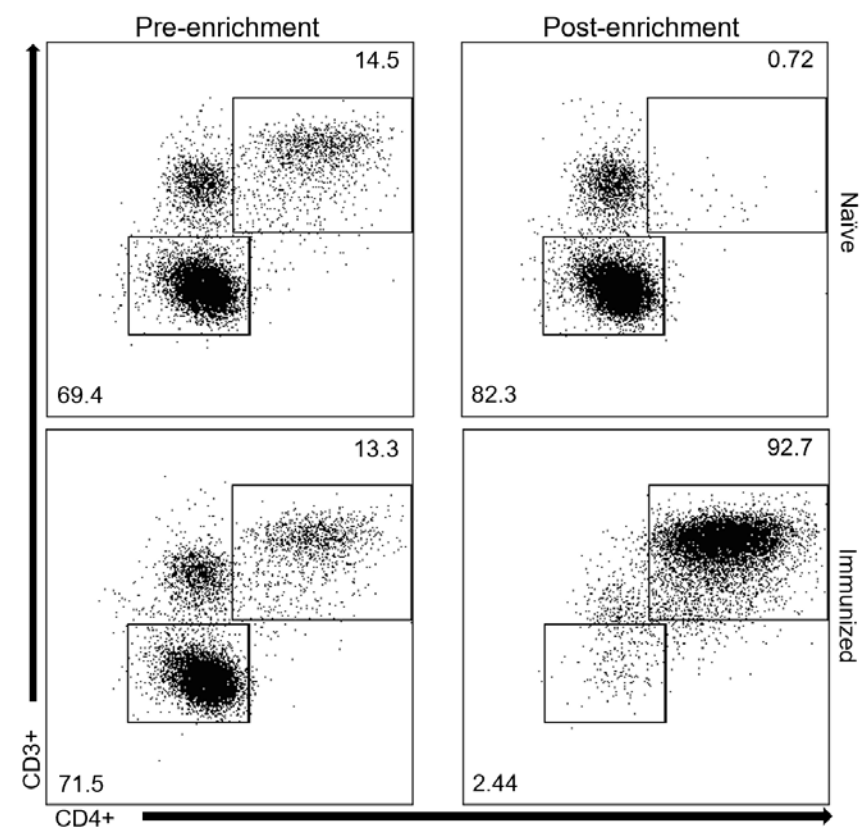

Supplementary Figure 2: **Purity of CD4$^+$ T cell and APC populations.** Pre-enrichment and post-enrichment evaluation of T cells in naïve and immunized mice. The CD45$^+$ population was gated on the single cell population within the live cell gate. The proportion of CD3$^+$CD4$^+$ T cells in this population was determined. One representative experiment of at least 4 independent experiments is shown.

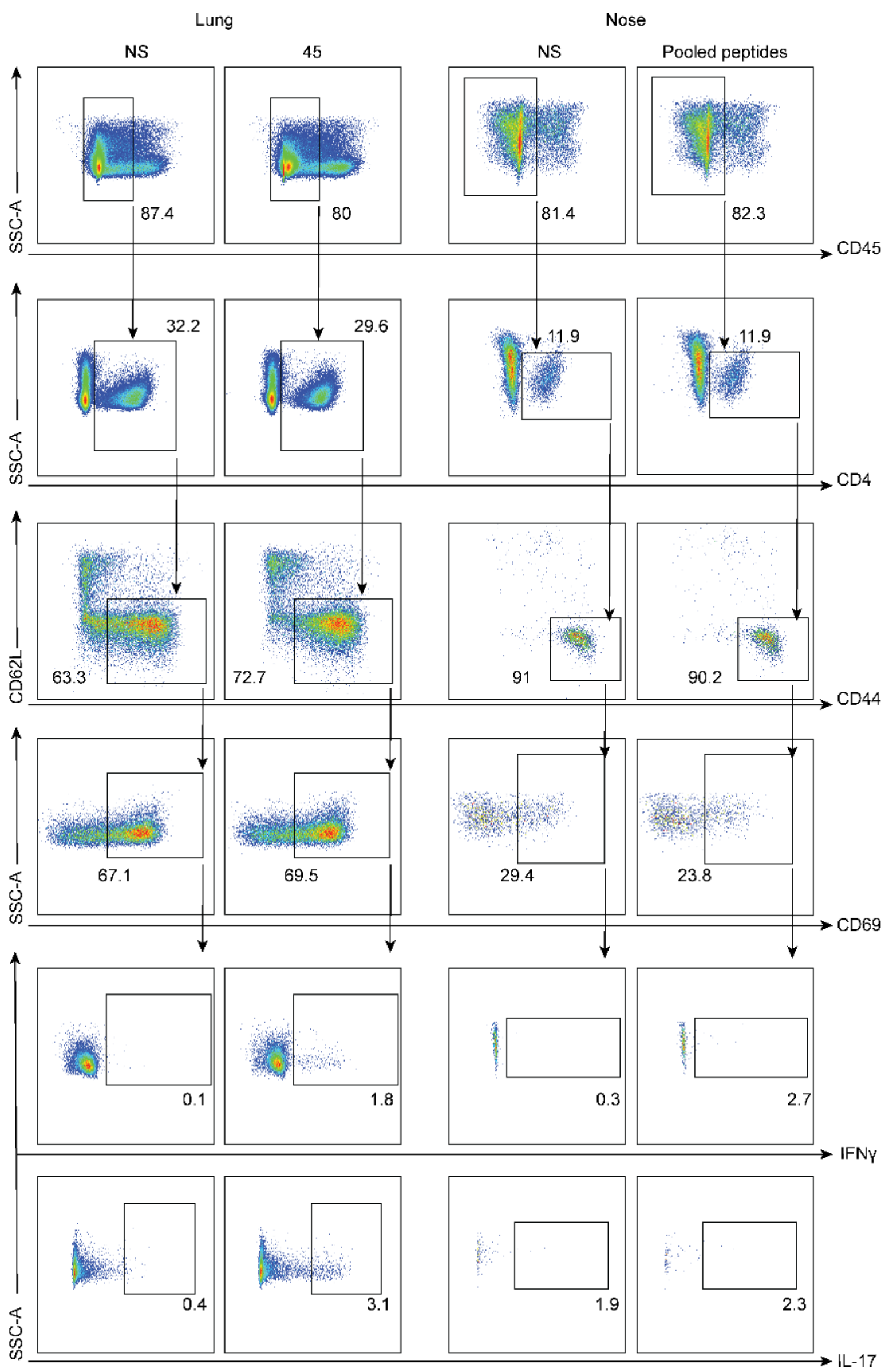

Supplementary Figure 3: Gating strategy to identify CD4$^+$ T$_{RM}$ in the lungs and nose of Bp536 and H762 infected mice. Shown is the sequential gating strategy to identify the target population: CD45$^-$ cells, CD4$^+$ cells, CD44$^+$CD62L$^-$ cells and CD69$^+$ cells. The proportion of IFNγ$^+$ and IL-17$^+$ cells within the CD4$^+$CD45$^-$CD44$^+$CD62L$^-$CD69$^+$ fraction was determined.

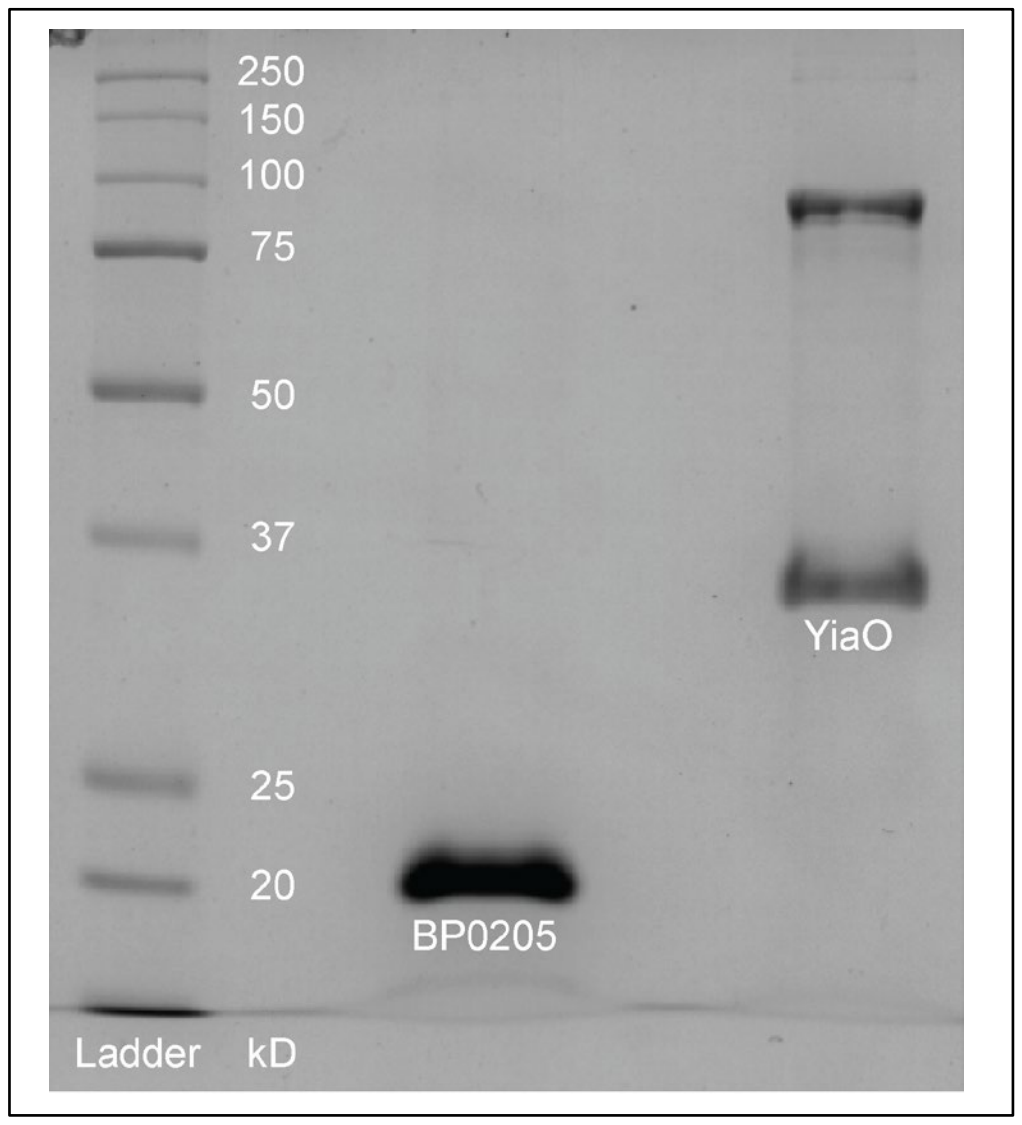

Supplementary Fig. 4: Recombinant proteins produced in *E. coli* are of the expected size.

BP0205 is a 19 kD single moiety while YiaO was detected as a 32 kD single moiety and putative 96 kD multimer.

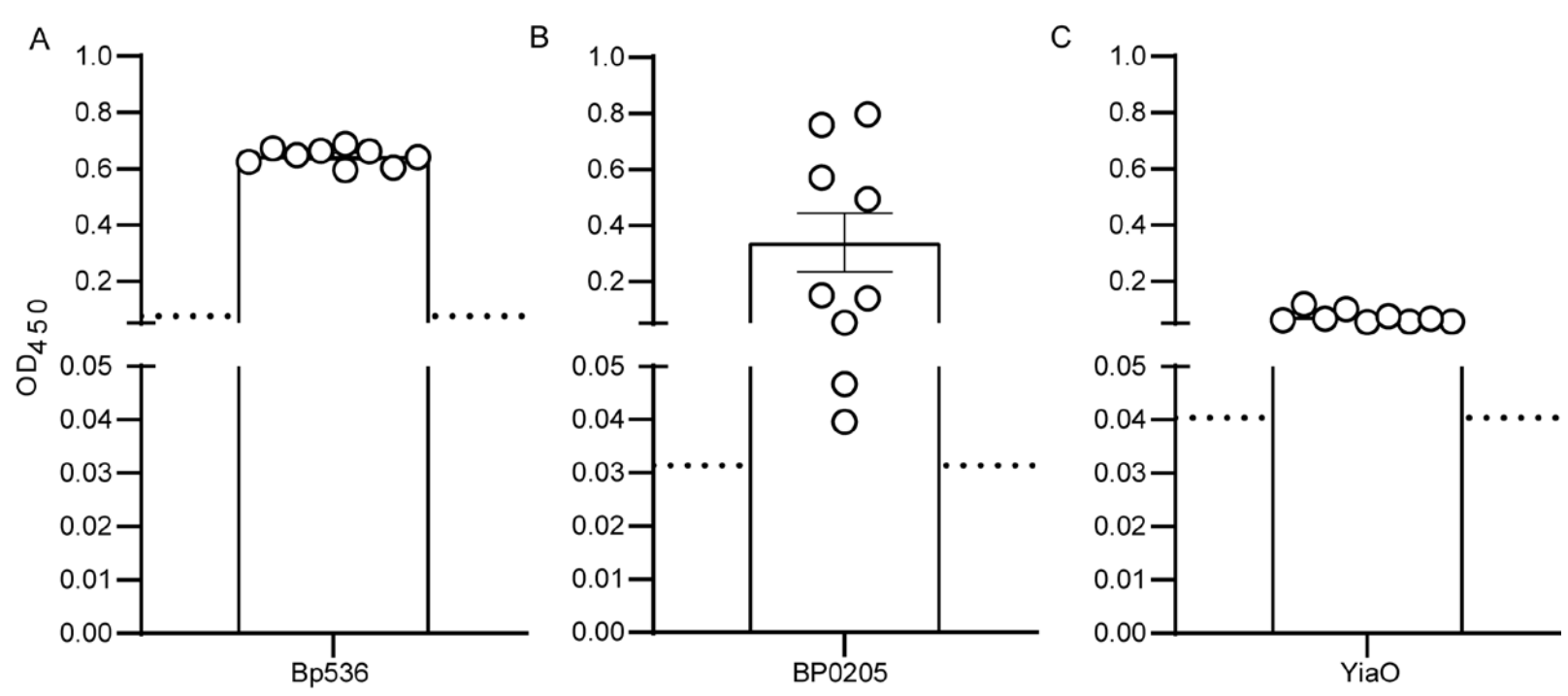

Supplementary Figure 5: Recognition of recombinant BP0205 and YiaO by serum of *Bp* convalescent mice.

Recognition of **(A)** Bp536 cell lysate **(B)** recombinant proteins BP0205 and **(C)** YiaO by convalescent sera from Bp536 challenged mice. Each circle denotes serum from an individual mouse. The dotted line denotes the average value of negative control wells containing buffer alone.

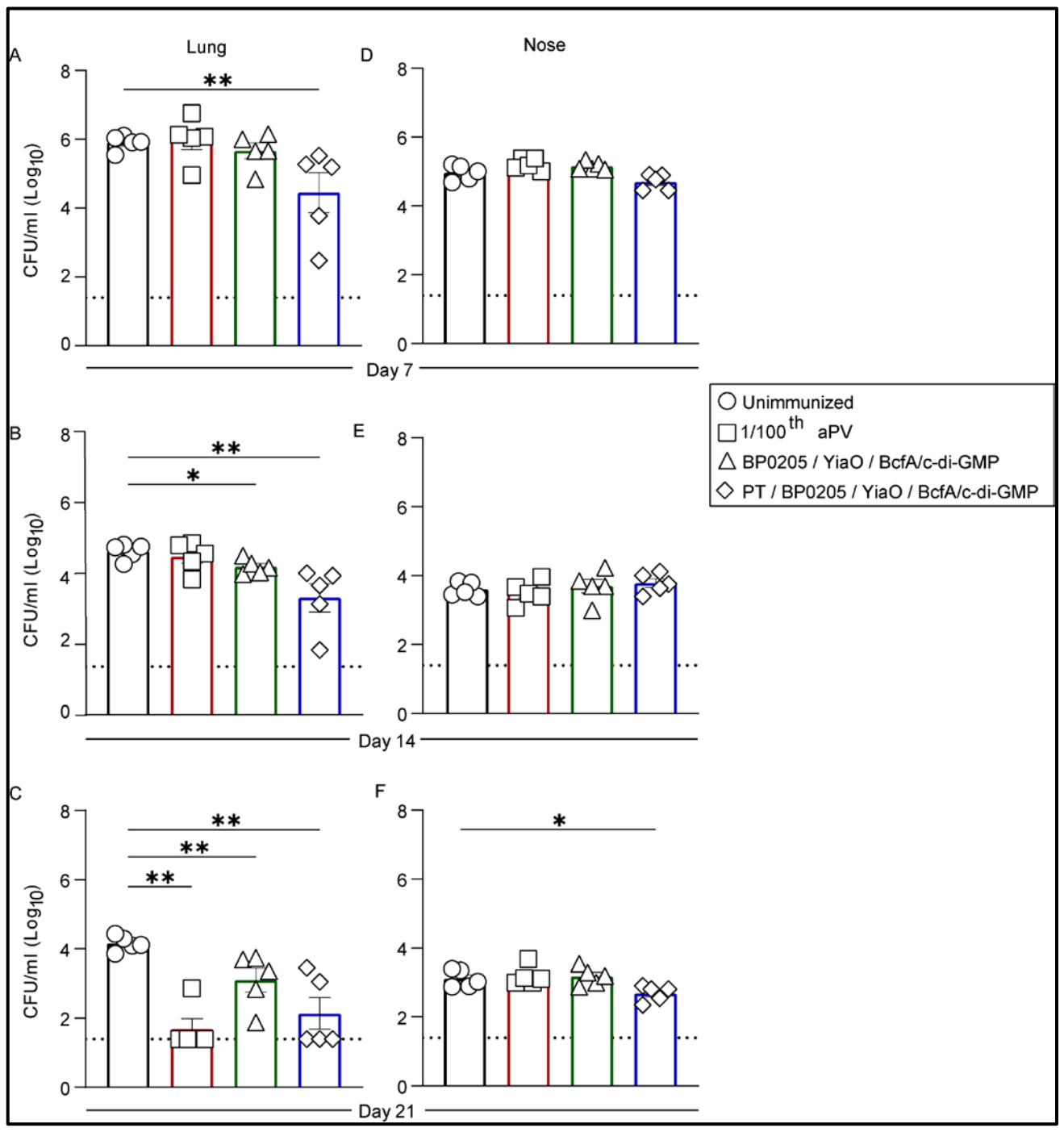

Supplementary Figure 6: Immunization with PT/BP0205/YiaO/BcfA/c-di-GMP reduces respiratory *Bp* burden with accelerated kinetics compared to aPV Boostrix™.

Immunized and unimmunized mice were harvested at the indicated days post-challenge. Colonization of the lungs on **(A)** day 7 **(B)** day 14 and **(C)** day 21 post-challenge. Colonization of the nose on **(D)** day 7 **(E)** day 14 and **(F)** day 21 post-challenge. $*P < 0.05$ and $**P < 0.01$ of the mean ± SEM compared to unimmunized mice by one way ANOVA with Dunnett's *post-hoc* test.

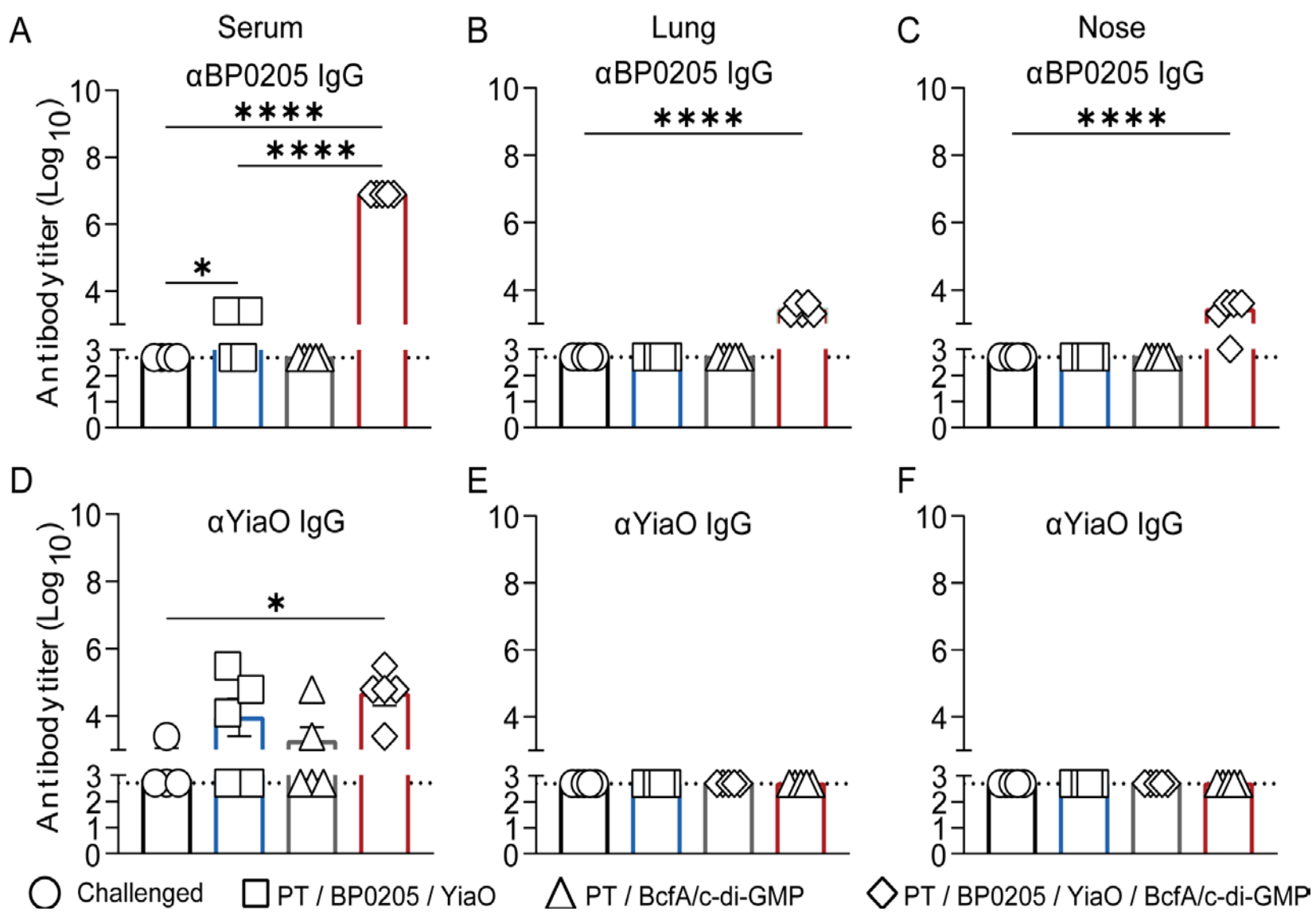

Supplementary Figure 7: Immunization with PT/BP0205/YiaO/BcfA/c-di-GMP elicits antigen specific IgG antibodies.

BP0205-specific IgG antibody titers in **(A)** serum, **(B)** lung and **(C)** nose, and anti-YiaO specific IgG antibodies in the **(D)** serum, **(E)** lung and **(F)** nose at day 7 post challenge. Data shown are mean ± SEM. One-way ANOVA with Dunnett's multiple comparisons test was used to compare groups. *P < 0.05 and ****P< 0.0001. The dotted line denotes the limit of detection.
