# Supplementary material for: Discovery of CD4+ T cell–recognized B. pertussis antigens that reduce airway colonization
Source: JCI Insight. 2026 Sep 8;11(17):e199234. doi: 10.1172/jci.insight.199234 (PMC13564080; doi:10.1172/jci.insight.199234)

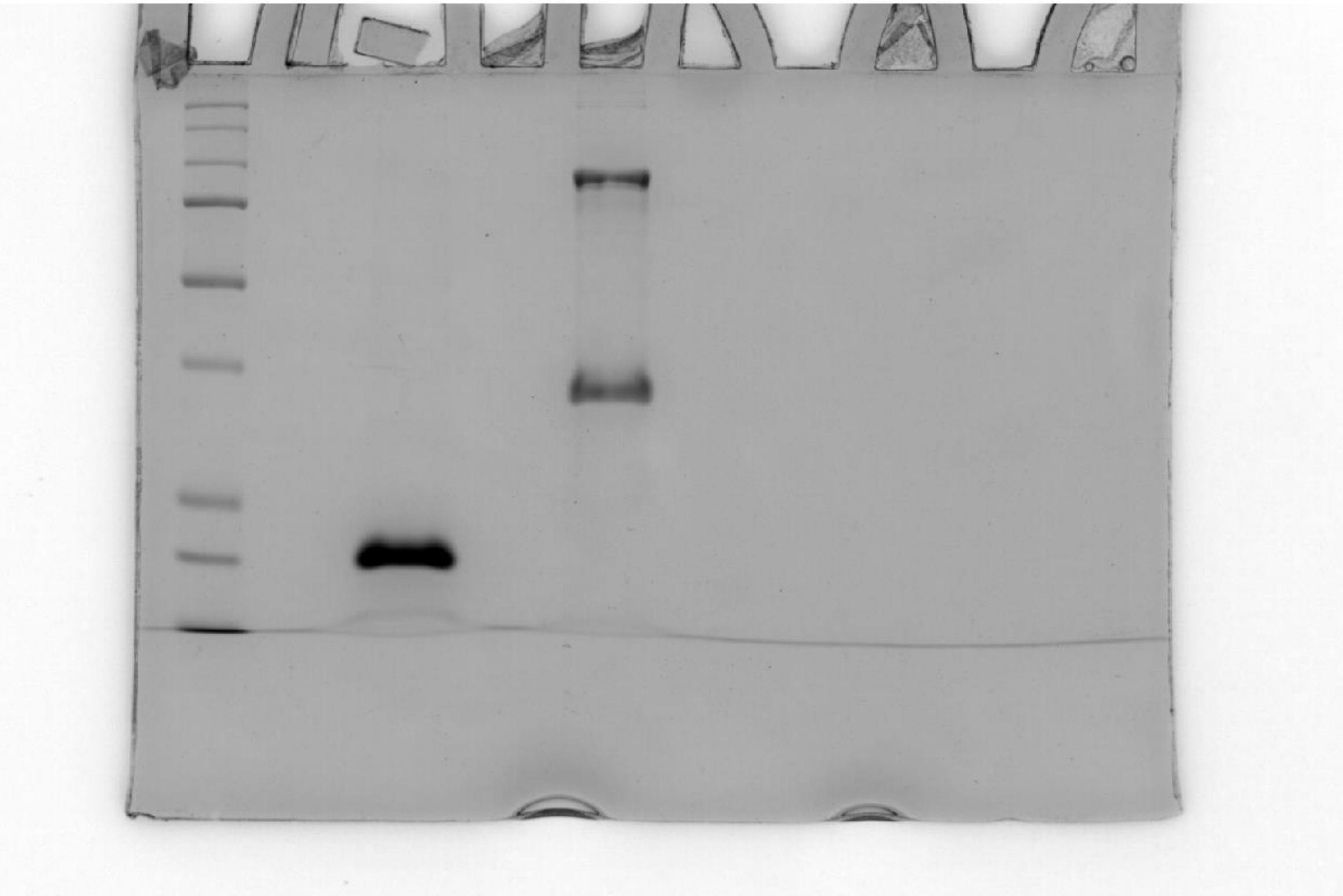

Supplement: Supplementary file 2 [file jciinsight-11-199234-s061.pdf]
